# Circulating lipoprotein (a) and all-cause and cause-specific mortality: a systematic review and dose-response meta-analysis

**DOI:** 10.1007/s10654-022-00956-4

**Published:** 2023-01-28

**Authors:** Mojgan Amiri, Hamidreza Raeisi-Dehkordi, Auke J.C.F Verkaar, Yahong Wu, Anniek C. van Westing, Kirsten A. Berk, Wichor M. Bramer, Dagfinn Aune, Trudy Voortman

**Affiliations:** 1 Department of Epidemiology, Erasmus MC, University Medical Center Rotterdam, Rotterdam, The Netherlands; 2 Institute of Social and Preventive Medicine (ISPM), University of Bern, Bern, Switzerland; 3 Julius Center for Health Sciences and Primary Care, University Medical Center Utrecht, Utrecht University, Utrecht, The Netherlands; 4 Division of Human Nutrition and Health, Wageningen University & Research, Wageningen, The Netherlands; 5 Department of Internal Medicine, Division of Pharmacology and Vascular Medicine, Erasmus MC, University Medical Center Rotterdam, Rotterdam, The Netherlands; 6 Department of Internal Medicine, Division of Dietetics, Erasmus MC University Medical Center, Rotterdam, The Netherlands; 7 Medical Library, Erasmus MC, University Medical Center Rotterdam, Rotterdam, The Netherlands; 8 Department of Epidemiology and Biostatistics, School of Public Health, Imperial College London, London, UK; 9 Department of Nutrition, Bjørknes University College, Oslo, Norway; 10 Department of Endocrinology, Morbid Obesity and Preventive Medicine, Oslo University Hospital, Oslo, Norway

**Keywords:** Mortality, Cause of death, Cardiovascular disease, Lipoprotein(a), Survival, Heart disease risk factors, Cohort studies, Chronic disease, Meta-analysis

## Abstract

**Aims:**

To investigate the association between circulating lipoprotein(a) (Lp(a)) and risk of all-cause and cause-specific mortality in the general population and in patients with chronic diseases, and to elucidate the dose-response relations.

**Methods and results:**

We searched literature to find prospective studies reporting adjusted risk estimates on the association of Lp(a) and mortality outcomes. Forty-three publications, reporting on 75 studies (957,253 participants), were included. The hazard ratios (HRs) and 95% confidence intervals (95%CI ) for the top versus bottom tertile of Lp(a) levels and risk of all-cause mortality were 1.09 (95%CI: 1.01–1.18, I^2^: 75.34%, n = 19) in the general population and 1.18 (95%CI: 1.04–1.34, I^2^: 52.5%, n = 12) in patients with cardiovascular diseases (CVD). The HRs for CVD mortality were 1.33 (95%CI: 1.11–1.58, I^2^: 82.8%, n = 31) in the general population, 1.25 (95%CI: 1.10–1.43, I^2^: 54.3%, n = 17) in patients with CVD and 2.53 (95%CI: 1.13–5.64, I^2^: 66%, n = 4) in patients with diabetes mellitus. Linear dose-response analyses revealed that each 50 mg/dL increase in Lp(a) levels was associated with 31% and 15% greater risk of CVD death in the general population and in patients with CVD. No non-linear dose-response association was observed between Lp(a) levels and risk of all-cause or CVD mortality in the general population or in patients with CVD (P_nonlinearity_ > 0.05).

**Conclusion:**

This study provides further evidence that higher Lp(a) levels are associated with higher risk of all-cause mortality and CVD-death in the general population and in patients with CVD. These findings support the ESC/EAS Guidelines that recommend Lp(a) should be measured at least once in each adult person’s lifetime, since our study suggests those with higher Lp(a) might also have higher risk of mortality.

**Supplementary information:**

The online version contains supplementary material available at 10.1007/s10654-022-00956-4.

## Introduction

Lipoprotein(a) (Lp(a)) consists of a cholesteryl-ester rich lipid core and an apolipoprotein B-100 bound to apolipoprotein(a) (apo(a)). Lp(a) has proatherogenic and prothrombotic properties and, is considered a risk factor for the development of cardiovascular disease (CVD) [1–6]. Also, European Society of Cardiology and the European Atherosclerosis Society (ESC/EAS) Guidelines for the Management of Dyslipidemias suggest to measure Lp(a), since it helps to identify people with high levels of Lp(a) that may have a higher risk of CVD [7].

Despite well-established evidence on Lp(a) as a risk factor of CVD, findings on relation between this marker and mortality are controversial. Also, this association has been studied more extensively in the general/healthy population than in patients. The Emerging Risk Factors Collaboration in 2009 investigated the association of Lp(a) concentration and risk of coronary heart disease, stroke, and mortality in healthy and general population. The findings of this individual participant meta-analysis of cohort and nested case-control studies indicated the risk ratios (RR) of 1.14 (95% Confidence interval (95%CI): 1.07–1.22, number of studies (n)= 24) and 1.01 (95%CI: 0.98–1.05, n= 25) for aggregate of coronary and nonvascular mortality [8]. Among publications on patients with chronic diseases, the Ludwigshafen Risk and Cardiovascular Health study in patients with prevalent coronary heart disease revealed no associations between Lp(a) concentrations and genetic variants with all-cause or CVD mortality [9]. Furthermore, analyses on plasma Lp(a) concentrations and risk of CVD death in two prospective cohorts of individuals with type 2 diabetes (T2DM) found no considerable association of this marker and CVD death [10]. On the contrary, other studies have found increased risk of mortality with elevated Lp(a) in the general population [11, 12] or in patients with CVD [13, 14] or T2DM [15].

Regardless of a considerable number of publications, few studies have summarized the association of circulating Lp(a) with the risk of mortality. The latest meta-analysis on this association was published in 2011, which investigated the association with all-cause mortality as a secondary outcome in the general population [16]. Another meta-analysis [17] focused only on patients with coronary artery diseases and used the highest versus lowest approach to perform the meta-analysis which is not the best approach as the highest and lowest values of Lp(a) may vary substantially between studies. None of the above-mentioned studies have presented the dose-response associations [16, 17]. In addition, findings of an individual patient meta-analysis of clinical trials on statin treated patients revealed approximately linear association between elevated baseline and on-statin Lp(a) levels and risk of cardiovascular diseases [18]. Other studies narratively reviewed the findings [19] or focused on a few number of studies represented by consensus panel or collaborations [1]. Moreover, it remains unclear whether levels of Lp(a) might be associated with the risk of mortality in a same pattern in the general population or in patients with chronic diseases. Thus, in the current study, we systematically reviewed the association between circulating Lp(a) and all-cause and/or cause-specific mortality either in the general population or in patients with chronic diseases. Additionally, we performed dose-response analyses to clarify the strength and shape of relationship between Lp(a) and mortality outcomes.

## Materials and methods

### Review design

This systematic review was designed and reported in accordance with PRISMA guidelines [20]. The study protocol was registered in the PROSPERO database on Nov 16, 2020 (registration number: CRD42020213420).

### Data sources and search strategy

Embase.com, Medline ALL (Ovid), Web of science Core Collection, and Cochrane Central were searched until June 8, 2021. Lipoprotein(a) and mortality-related keywords were combined in the search strategy. Furthermore, filters to exclude conference abstracts, case-report studies, non-adult populations, and animal studies were applied. The search strategy was developed by an expert research librarian (WB). The search results were imported in EndNote and de-duplicated with the method as described by Bramer et al. [21]. The details of the search strategy and keywords are presented in **Supplementary Table-1**. To complete our search, the references of the included studies and published reviews were manually reviewed.

### Eligibility criteria

We included all original prospective observational investigations, including cohort and case-cohort studies, reporting adjusted risk estimates of the association of circulating Lp(a) with all-cause or cause-specific mortality in adults (≥ 18 years) irrespective of health and diseases status. No date restrictions were applied.

Retrospective studies, conference abstracts, ecological studies, case reports, case-series, letters to the editor, conference proceedings, narrative reviews, systematic reviews or meta-analyses as well as studies conducted in animals, children, or adolescents were excluded. We did not include non-English language articles.

It should be noted, to provide a complementary report of all available evidence of this association, the studies reporting unadjusted and/or descriptive/frequency results are also summarized in the **Supplementary Tables-4 and 5**.

### Study selection, data extraction, and quality assessment

All titles/abstracts were screened in duplicate by two independent groups of researchers (MA and HRD together with AV, YW, AVW, and KB) according to the eligibility criteria. Afterward, all provided full-texts were reviewed similarly in duplicate as the previous step. MA and HRD extracted data from the included studies based on a predesigned form. The main extracted information from the included studies was the first author’s name, study design, publication year, location, number of participants, sex distribution of the population, participants’ health status at study entry, age, follow-up duration, Lp(a) assessment method, number of deaths, causes of mortality, adjustments, and hazard/ risk/ odds ratios.

Quality of the included studies was assessed using Newcastle-Ottawa scale [22] by MA and HRD, independently. Each study was judged based on selection, comparability, and outcome/exposure domains. Studies which achieved fewer than four points, four to six points, and seven or more points were graded as poor, fair and good quality. Discrepancies were solved through discussion and unsettled disagreements were arbitrated by the third investigator (TV).

### Data analysis

We performed DerSimonian-Laird random effects model to calculate the pooled hazard ratios (HR) and corresponding 95% confidence intervals (95%CI) [23]. For studies with reported odds ratio (OR) between 0.5 and 2.5 or relative risks (RR), values were treated as the HR [24, 25] and risk estimates of the most adjusted model were extracted. To enable a consistent and standardized approach in the meta-analysis, all risk estimates for the association between Lp(a) and mortality were transformed to top versus bottom third of Lp(a) distribution in each study [26]. Further details on the risk conversion methods are provided the following section. For a study [27] that used other categories than the first one as the reference category, we changed the reference category to the lowest one using the method developed by Hamling [28]. We calculated standard errors (SE) using the method developed by Greenland [29] for studies that did not report CI or SE [30–33]. Before inclusion in the meta-analysis, the risk estimates of a study [34] providing results stratified by gender, but not overall, were pooled using a fixed-effects model.

Heterogeneity between studies was assessed using the I^2^ [35]. We conducted subgroup analyses by region (Asia, Europe, America, Australia, or multiple regions combined), follow-up duration (< 10 or ≥ 10 years), by whether studies adjusted for body mass index (BMI) or lipid lowering medication intake as confounders, and Lp(a) assessment methods. Additionally, among the included studies, there were some articles that were conducted on more than one cohort but did not report the association of Lp(a) and mortality in each cohort separately [8, 10, 11, 36]. Therefore, we performed a subgroup analysis stratified by whether papers included multiple studies (Yes/No). We also conducted a sensitivity analysis to explore if the results were robust using leave-one-out analysis, excluding each study at a time from analysis. Publication bias was assessed with Egger’s test and asymmetry was visually explored with funnel plots [37, 38].

To perform the linear dose-response analyses, the method by Greenland and Longnecker [39] was used. Using this method, we calculate the HRs and 95%CI from the natural logarithm of the risk estimated across the categories of Lp(a). We considered HRs for risk of mortality per 50 mg/dL Lp(a) increase, since it has been suggested that lowering of Lp(a) by 50 mg/dL may reduce the risk of CVD [40]. Also, the findings of studies on the association of extreme levels of Lp(a) with risk of CVD showed a stepwise increase in risk of CVD with no evidence of a threshold effect [41–43] and one of the biggest studies in the current systematic review performed by Langsted et al. [11] presented the HRs per 50 mg/dL increase in Lp(a) for risk of mortality (all-cause, cardiovascular and non-vascular mortality). Additionally, a nonlinear dose-response association between Lp(a) and mortality was assessed using restricted cubic splines with three knots at 10%, 50% and 90% percentiles of the distribution, which was combined using multivariate meta-analysis [44, 45]. We included studies that reported the risk of mortality for at least three categories of Lp(a) in these analyses.

We performed the statistical analyses of our meta-analysis in R (Version 4.0.5) using meta and dmetar packages and Stata (Version 16.0) was used to perform the dose-response analyses.

### Risk conversions

For studies that reported the mortality in tertiles of Lp(a), the HRs or RRs were included as reported. For studies that grouped the exposure in quartile, quintile, high versus low level of the marker, or reported the results as continuous, such as per SD or per unit, we converted the HRs or RRs from the original studies to the HRs or RRs for the third versus bottom tertile of Lp(a) distribution using defined methods [26]. Based on this method, to convert log relative risks from the reported scale in the studies to top versus bottom third, we need to use conversion factors driving based on the ratio of expected differences in mean levels of the standardized exposure, for the target comparison versus reported comparison.

For example, a factor of 2.54 is the difference in the means of the upper and lower quartiles and the expected difference in means of the top versus bottom thirds of the standard normal distribution is 2.18. So, by applying a multiplication conversion factor of 2.18/2.54 to the log relative risk and its standard error, we can convert a top versus bottom quartile comparison to correspond to the top versus bottom third comparison. Similarly, a conversion factor of 2.18 and 2.18 * SD are used to obtain the risk for the third versus first tertile for the estimations reported per SD and unit increment of exposure, respectively. The multiplicative conversion factors of 2.18/2.80 and 2.18/1.59 were used to transform the RR for the top versus the bottom quintiles and high versus low levels of Lp(a). This method has been widely used in previous publications [46–50].

## Results

### Eligible studies and characteristics

Our search returned 2,133 references. Out of 2,133 titles and abstracts, 256 articles were qualified for a full text screening. From these 256, we excluded articles with irrelevant outcomes (n = 118) or study design (n = 28) and those with non-English languages (n = 3). Additionally, 8 articles did not investigate Lp(a) as the exposure and 8 articles were excluded due to the identical populations to the other included studies. One duplicate title and one article with irrelevant population were also excluded. Furthermore, to avoid double-counting of the included studies, we excluded those studies (n = 2) that overlapped with the Risk Factors Collaboration study [8]. We could not retrieve the full-texts of 8 titles.

**Fig. 1:**
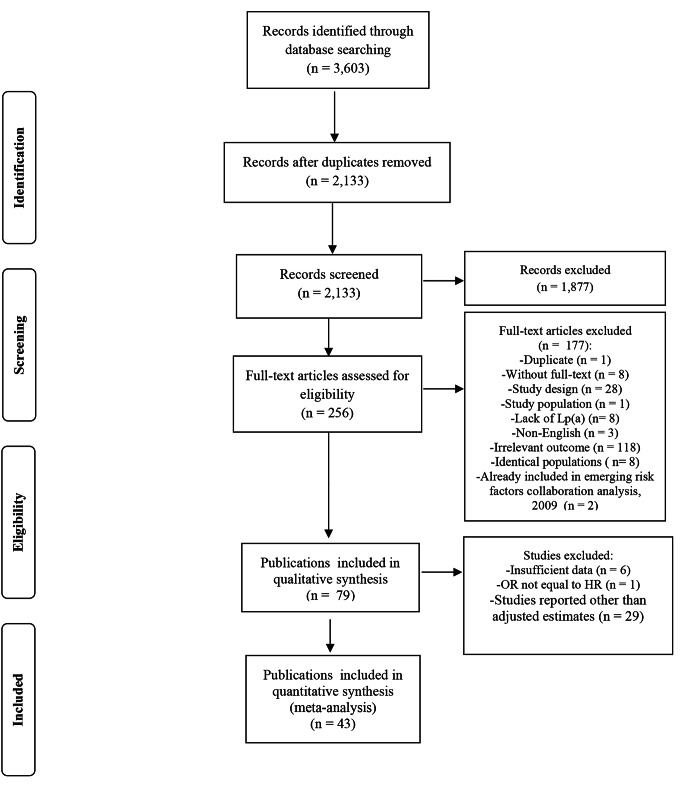
Study selection procedure

Hence, 79 papers were further assessed. Of these, 29 studies reported unadjusted results as either univariate risks, mortality number/rate in different levels of Lp(a), or level of Lp(a) in survivors and non-survivors, which were excluded from our analyses but their key characteristics and results are summarized in **Supplementary Tables-4 and 5**. 4 studies [51–54] and subgroup analysis of patients with DM from one of the included studies [36] were excluded due to insufficient data to perform the risk conversions; 2 studies used another category of Lp(a) than the first as the reference category and did not report enough information to apply Hamling method [55, 56]; and one study [57] reported OR which values could not be treated as HRs. Thus, 43 publications were included in our meta-analysis. Among these publications, 4 articles reported the pooled results of participants from 7 [36], 2 [11], 2 [10], 25 [8] studies, leading to 75 studies and 957,253 individuals. Figure 1 represents the study selection procedure.

**Supplementary Table-2** presents the main characteristics of the included studies in the meta-analysis. Briefly, these studies have been published between 1998 and 2020. The association of Lp(a) concentration and all-cause or cause-specific mortality was reported in general or healthy populations [8, 11, 12, 34, 36, 58–70], individuals with CVD [13, 14, 30, 67, 69, 71–84], chronic renal failure (CRF) [27, 31–33], and DM [10, 15, 67, 85, 86]. One study was conducted in women only [78] and two in men only [64, 68]. The follow-up duration ranged from 1 to 20 years.

Additionally, 20 studies were graded as fair and the remaining as good quality, according to Newcastle – Ottawa quality assessment scale, as presented in **Supplementary Table-3.**

### Meta-analysis and dose-response analysis

#### All-cause mortality

The association of Lp(a) and all-cause mortality is summarized in Table 1. Higher levels of Lp(a) was associated with a higher risk of all-cause mortality in the general population (HR: 1.09, 95%CI: 1.01–1.18, I^2^: 75.34%, n = 19, **Supplementary Figure-1**) and in patients with CVD (HR: 1.18, 95%CI: 1.04–1.34, I^2^: 52.5, n = 12, **Supplementary Figure-2**). No significant association was found in patients with CRF or DM. The results of meta-analysis were not robust in leave-one-out analysis in general population by removing several effect sizes [11, 12, 34, 62, 63, 65–67], but in patients with CVD, the association remained unchanged (**Supplementary Figure-6-A and B**). Funnel plots showed no asymmetry in both populations (Egger’s p-value > 0.05, **Supplementary Figure-7-A and B**).

**Table 1:** Summary risk estimates and stratified analyses for association between circulating Lp(a) and all-cause mortality in general population, patients with CVD, CRF or DM

	No. of effect sizes	No. of studies	No. of participants	Pooled HR (95%CI)	I^2^%	p-value (between groups)
General population						
Overall effect	12	19	169,432	1.09 (1.01, 1.18)	75.34	-
Region						
*Asia*	3	3	15,171	1.24 (0.81, 1.89)	86.8	0.63
*Europe*	7	14	149,056	1.05 (0.96, 1.14)	74.9	
*America*	2	2	5,205	1.09 (0.99, 1.22)	0	
Follow-up duration						
*< 10 years*	8	14	67,054	1.04 (0.94, 1.16)	54.8	0.36
*≥ 10 years*	4	5	102,378	1.15 (0.96, 1.37)	89.7	
Multiple studies^*^						
*Yes*	2	9	121,892	0.99 (0.92, 1.06)	70.8	0.05
*No*	10	10	47,540	1.13 (1.01, 1.27)	67.0	
Adjustment for BMI						
*Yes*	9	16	162,669	1.07 (0.99, 1.16)	79.6	0.34
*No*	3	3	6,763	1.30 (0.87, 1.95)	30.5	
Adjustment for Lipid-lowering medication						
*Yes*	3	9	58,294	1.06 (0.88, 1.27)	74.0	0.72
*No*	9	10	111,138	1.10 (0.98, 1.23)	77.7	
Lp(a) assessment methods						
*ELISA*	6	6	20,033	1.07 (0.88, 1.29)	75.9	0.22
*ITA*	3	9	73,434	1.08 (0.92, 1.27)	87.3	
*INA*	1	1	1,274	1.32 (0.82, 2.12)	-	
*NM*	1	1	4,930	1.78 (1.02, 3.09)	-	
*Multiple methods*	1	2	69,761	1.02 (1.005, 1.03)	-	
**Patients with CVD**						
Overall effect	12	12	19,762	1.18 (1.04, 1.34)	52.5	-
Region						
*Asia*	4	4	7,255	1.34 (1.04, 1.72)	44.6	0.67
*Europe*	6	6	9,415	1.14 (0.87, 1.48)	65.3	
*America*	1	1	1,620	1.14 (1.01, 1.27)	-	
*Australia*	1	1	1,472	1.11 (0.82, 1.50)	-	
Follow-up duration						
*< 10 years*	11	11	18,798	1.19 (1.04, 1.37)	56.6	0.50
*≥ 10 years*	1	1	964	1.06 (0.76, 1.47)	-	
Adjustment for BMI						
*Yes*	5	5	12,524	1.12 (0.98, 1.27)	47.3	0.35
*No*	7	7	7,238	1.28 (0.98, 1.68)	58.0	
Adjustment for Lipid-lowering medication						
*Yes*	4	4	11,535	1.08 (0.98, 1.18)	18.8	0.09
*No*	8	8	8,227	1.34 (1.05, 1.72)	54.0	
Lp(a) assessment methods						
*ELISA*	4	4	5,484	1.27 (1.006, 1.61)	53.1	0.04
*ITA*	3	3	5,571	1.24 (1.03, 1.50)	23.1	
*INA*	2	2	2,956	0.89 (0.48, 1.64)	62.7	
*IRMA*	1	1	966	2.59 (1.19, 5.62)	-	
*Automated latex enhanced immunoassay*	1	1	1,472	1.11 (0.82, 1.50)	-	
*Photometric assay*	1	1	3,313	0.95 (0.81, 1.11)	-	
**Patients with CRF**						
Overall effect	3	3	4,188	1.96 (0.89, 4.32)	65.4	-
**Patients with DM**						
Overall effect	2	2	596	0.97 (0.72, 1.32)	0.0	-

^*^ Some publications were conducted on more than one cohort study without reporting the separated results. CVD: Cardiovascular disease; CRF: Chronic renal failure; DM: Diabetes mellitus; HR: Hazard ratio; 95%CI: 95% confidence interval; ELISA: Enzyme-linked immunosorbent assay; ITA: Immunoturbidimetric assay; INA: Immunonephelometric assay; NM: Not mentioned

**Fig. 2:**
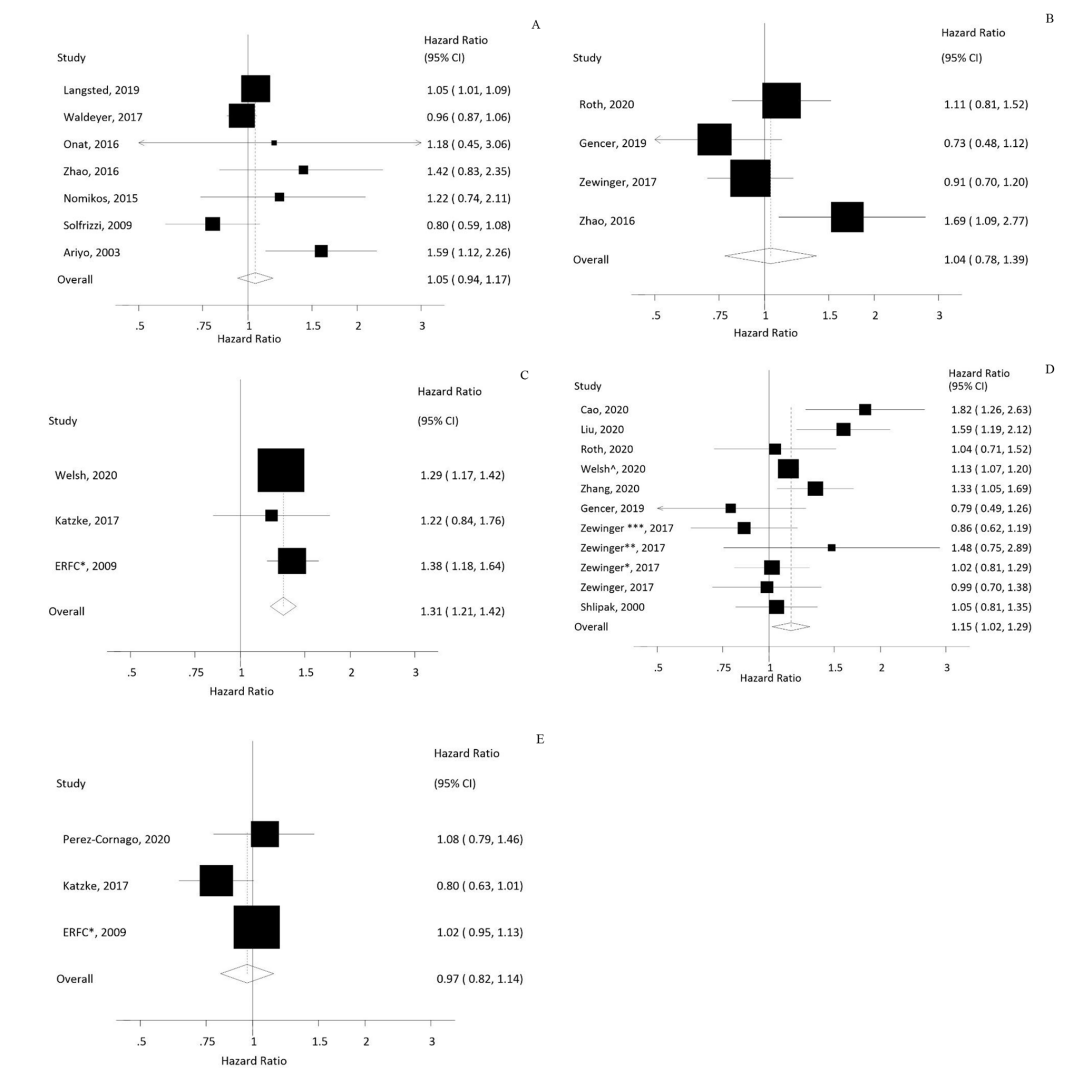
Linear dose-response analysis of Lp(a) per 50 mg/dL and **A**) All-cause mortality in general population, **B**) All-cause mortality in patients with CVD, **C**) CVD- death in general population, **D**) CVD-death in patients with CVD, **E**) Non-CVD-death in general population. ^*^ERFC: The emerging risk factor collaboration. Zwinger^*^: Validation study 1; Zwinger^**^: Validation study 2; Zwinger^***^: Validation study 3; Welsh^^^: High risk cohort

Moreover, there was no significant linear dose-response association either in general population (HR: 1.05, 95%CI: 0.94–1.17, I^2^: 54.6%, n = 14, Fig. 2 A) or among patients with CVD (HR: 1.04, 95%CI: 0.78–1.39, I^2^: 61.7%, n = 4, Fig. 2B). Also, no non-linear dose-response association was shown between Lp(a) and all-cause mortality in these populations (p_nonlinearity_ > 0.05, **Supplementary Figure-8-A and B, Supplementary Table-6**).

#### CVD and non-CVD deaths

Table 2 summarizes the risk estimates for association between circulating Lp(a) and CVD-death. Our meta-analysis indicated an association of Lp(a) with higher risk of CVD-death in general population (HR: 1.33, 95%CI: 1.11–1.58, I^2^: 82.8%, n = 31, **Supplementary Figure-3**), patients with CVD (HR: 1.25, 95%CI: 1.10–1.43, I^2^: 54.3%, n = 17, **Supplementary Figure-4**) and patients with DM (HR: 2.53, 95%CI: 1.13–5.64, I^2^: 66%, n = 4). These results were robust in leave-one-out analysis (**Supplementary Figure-6-C and D**) and no asymmetry was detected in funnel plots (Egger’s p-value > 0.05, **Supplementary Figure-7-C and D**).

**Table 2:** Summary risk estimates and stratified analyses for association between circulating Lp(a) and CVD-death in general population, patients with CVD, CRF or DM

	No. of effect sizes	No. of studies	No. of participants	Pooled HR (95%CI)	I^2^%	p-value (between groups)
General population						
Overall effect	8	31	112,157	1.33 (1.11, 1.58)	82.8	-
Region						
*Asia*	1	1	10,413	1.45 (0.91, 2.31)	-	0.93
*Europe*	6	6	29,061	1.33 (1.04, 1.69)	87.4	
*Multiple regions combined*	1	24	72,683	1.33 (1.15, 1.54)	-	
Follow-up duration						
*< 10 years*	5	28	80,285	1.21 (1.03, 1.44)	74	0.36
*≥ 10 years*	3	3	31,872	1.45 (1.03, 2.05)	74.1	
Multiple studies^*^						
*Yes*	1	24	72,683	1.33 (1.15, 1.54)	-	0.96
*No*	7	7	39,474	1.34 (1.07, 1.67)	85	
Adjustment for BMI						
*Yes*	5	28	106,328	1.30 (1.003, 1.69)	88.8	0.47
*No*	3	3	5,829	1.70 (0.86, 3.35)	55.7	
Adjustment for Lipid-lowering medication						
*Yes*	2	2	7,669	1.81 (0.56, 5.84)	79.4	0.61
*No*	6	29	104,488	1.32 (1.09, 1.60)	86	
Lp(a) assessment methods						
*ELISA*	2	2	10,972	1.47 (0.95, 2.28)	0	0.01
*ITA*	2	2	19,060	1.48 (1.01, 2.16)	94.8	
*IRMA*	1	1	1,773	0.99 (0.87, 1.13)	-	
*NM*	2	2	7,669	1.80 (0.56, 5.84)	79.4	
*Multiple methods*	1	24	72,683	1.33 (1.15, 1.54)	-	
**Patients with CVD**						
Overall effect	17	17	57,019	1.25 (1.10, 1.43)	54.3	
Region						
*Asia*	3	3	12,434	1.62 (1.33, 2.96)	0.0	0.03
*Europe*	12	12	42,719	1.14 (0.99, 1.30)	47.9	
*America*	1	1	1,383	1.33 (0.65, 2.72)	-	
*Multiple regions combined*	1	1	483	1.39 (0.37, 5.23)	-	
Follow-up duration						
*< 10 years*	16	16	52,857	1.29 (1.12, 1.48)	55.4	0.09
*≥ 10 years*	1	1	4,162	1.02 (0.80, 1.29)	-	
Adjustment for BMI						
*Yes*	9	9	20,993	1.15 (0.97, 1.36)	53.9	0.06
*No*	8	8	36,026	1.56 (1.18, 2.06)	57.9	
Adjustment for Lipid-lowering medication						
*Yes*	9	9	50,862	1.23 (1.03, 1.47)	61.5	0.52
*No*	8	8	6,157	1.38 (1.03, 1.84)	50.8	
Lp(a) assessment methods						
*ELISA*	4	4	3,062	1.78 (1.24, 2.57)	4.8	0.04
*ITA*	7	7	44,379	1.25 (1.06, 1.47)	66	
*INA*	2	2	2,956	0.89 (0.55, 1.45)	23.4	
*IRMA*	1	1	966	2.59 (1.04, 6.45)	-	
*Immunoassay method*	1	1	483	1.39 (0.37, 5.23)	-	
*Photometric assay*	1	1	3,313	0.99 (0.81, 1.20)	-	
*Latex agglutination assay*	1	1	1,860	1.31 (0.85, 2.02)	-	
**Patients with CRF**						
Overall effect	2	2	658	1.83 (0.19, 17.96)	89.3	
**Patients with DM**						
Overall effect	3	4	4,070	2.53 (1.13, 5.64)	66	

^*^ Some publications were conducted on more than one cohort study without reporting the separated results. CVD: Cardiovascular disease; CRF: Chronic renal failure; DM: Diabetes mellitus; HR: Hazard ratio; 95%CI: 95% confidence interval; ELISA: Enzyme-linked immunosorbent assay; ITA: Immunoturbidimetric assay; INA: Immunonephelometric assay; IRMA: Immunoradiometric assay; NM: Not mentioned

According to the findings of subgroups analyses in patients with CVD, higher levels of Lp(a) was associated with a higher risk of mortality in Asian studies (HR: 1.62, 95%CI: 1.33–2.96, I^2^: 0.0%) compared to the other regions (p-value between groups < 0.05).

In general population and patients with CVD, linear dose-response analysis revealed a higher risk of CVD-death with higher levels of Lp(a). A 50 mg/dL increase in Lp(a) concentration was associated with 31% and 15% greater risk of CVD-death in both general population and patients with CVD (HR: 1.31, 95%CI: 1.21–1.42, I^2^: 0%, n = 26 in the general population; HR: 1.15, 95%CI: 1.02–1.29, I^2^: 52.3%, n = 11 in patients with CVD, **Figure-2-C and D**). No non-linear dose response association was observed (p_nonlinearity_ > 0.05, **Supplementary Figure-8-C and D, Supplementary Table-6**).

We found no significant association between Lp(a) concentration and non-CVD-death in the general population (HR: 1.05, 95%CI: 0.91–1.21, I2: 68.4%, n = 29, **Supplementary Figure-5**, Table 3). No asymmetry was detected and the effect estimate was robust in sensitivity analysis (**Supplementary Figure-6-E and 2-E**). Also, no significant linear (HR: 0.96, 95%CI: 0.79–1.18, I^2^: 57.1%, n = 28, Fig. 2E) or non-linear dose response associations were found (p_nonlinearity_ > 0.05, **Supplementary Figure-8-E, and Supplementary Table-6**).

**Table 3:** Summary risk estimates and stratified analyses for association between circulating Lp(a) and non-CVD-death in the general population

	No. of effect sizes	No. of studies	No. of participants	Pooled HR (95%CI)	I^2^%	p-value (between groups)
General population						
Overall effect	6	29	119,121	1.05 (0.91, 1.21)	68.4	-
Region						
*Asia*	1	1	10,413	1.71 (1.20, 2.43)	-	0.01
*Europe*	3	3	4,676	0.95 (0.78, 1.15)	61.3	
*America*	1	1	1,764	1.87 (0.73, 4.80)	-	
*Multiple regions combined*	1	24	102,268	1.02 (0.95, 1.10)	-	
Follow-up duration						
*< 10 years*	3	26	104,196	1.02 (0.96, 1.10)	0.0	0.83
*≥ 10 years*	3	3	14,925	1.06 (0.77, 1.48)	85.5	
Multiple studies^*^						
*Yes*	1	24	102,268	1.02 (0.95, 1.10)	-	0.52
*No*	5	5	16,853	1.10 (0.86, 1.42)	74.8	
Adjustment for lipid-lowering medication						
*Yes*	1	1	2,739	0.77 (0.61, 0.97)	-	0.008
*No*	5	28	116,382	1.10 (0.96, 1.25)	59.6	

^*^ Some publications were conducted on more than one cohort study without reporting the separated results. HR: Hazard ratio; 95%CI: 95% confidence interval

## Discussion

This is the first dose-response meta-analysis that provides a comprehensive overview of the associations between circulating Lp(a) and all-cause and cause specific mortality in the general population and in patients with chronic diseases.

The association of Lp(a) and mortality might be explained by some pathways. Several meta-analyses have reported the association between Lp(a) and cardiovascular disease/events [17, 87]. In addition, large genetic and epidemiological studies have indicated that higher levels of Lp(a) is a causal risk factor for myocardial infarction [2], coronary disease [4], atherosclerosis stenosis [88], aortic-valve calcification and aortic valve stenosis [3, 5, 89], which some mechanisms related to these diseases might mediate the association of higher Lp(a) levels and increased risk of all-cause and CVD mortality. Some other studies shed light on the prothrombotic effects [90] and anti-fibrinolytic roles [91] of Lp(a) as well. Lp(a) may interfere with plasminogen activity through molecular similarity. The homology between the fibrinolytic proenzyme, plasminogen, and Apolipoprotein (a) (apo(a)), a unique protein component of Lp(a) without any fibrinolytic activity, may slow down fibrinolysis and indirectly induce thrombosis [92]. Furthermore, elevated levels of Lp(a), particularly its apo(a) fragment, may induce vascular inflammation [93, 94], leading to the progression of atherosclerosis [94, 95], which may have been associated with increased risk of CVD and its related mortality either independently or interactively [96].

The significant increase of the Lp(a)-associated risk for CVD death in the general population in our study is in line with the results of a study conducted by the Emerging Risk Factors Collaboration including 72,683 individuals from 24 studies, found that a one standard deviation (SD) (3.5 fold) higher Lp(a) was associated with 14% increase in CVD-death, while no significant association was observed in risk of non-vascular mortality [8]. Findings of the BiomarCaRE consortium showed no significant association between Lp(a) and all-cause mortality for Lp(a) levels ≥ 90th percentile in comparison to Lp(a) levels in the lowest third which is in contrast to our findings [36]. Similarly, a large prospective study of 3,313 patients with established coronary heart disease found no significant associations between highest versus lowest tertile of Lp(a) and risk of all-cause and CVD mortality [82]. Two prospective cohorts on 2,308 patients with T2DM from Nurses’ Health Study and the Health Professional Follow-Up Study (n = 2308) reported no significant association of coronary heart disease, and CVD per 1-SD higher log-transformed Lp(a), while a marginally significant association was observed for CVD mortality [10].

This study has some limitations that should be considered while interpreting its results. Although we performed our analysis separately for studies in the general population, some studies in this population might not exclude patients with pre-existing diseases. Thus, this act may provide bias induced by pre-existing diseases. Residual confounding could have affected the results since we performed a meta-analysis of observational studies. However, there was little indication of heterogeneity between subgroups that adjusted for BMI or lipid-lowering medication, we could not perform subgroup analysis based on other confounding variables including hypertension status, blood lipid markers, physical activity and smoking as the majority of included studies presented these variables in their multivariable statistical models. Since most studies lacked multiple Lp(a) assessments, the reported relative risks in the original studies may have been subject to underestimation due to regression dilution bias and random measurement error [97]. We observed a high heterogeneity among the included studies which might be derived from variations in sample sizes or in concentration of Lp(a) in different populations (such as different races and ethnicities), Lp(a) assessment methods, and adjustments for confounding variables. Regarding the Lp(a) assessment methods it should be noted that there are both differences in assay methods between studies and also varieties within each assessment category that might be a reason of the large heterogeneity. Also, the majority of the studies did not take the variability of number of kringle-IV type 2 (KIV2)-like domains into account. Number of KIV2-like domains are associated with the size of apo(a) in structure of Lp(a), which means alleles with fewer repeats of KIV2 encode smaller apo(a) isoforms [98, 99], associating with higher plasma Lp(a) concentrations. This fact also might be a reason for heterogeneity among the studies. We were unable to perform sex-stratified analysis and also subgroup analysis for patients with CVD based on disease clinical presentation (acute and chronic coronary syndrome) owing to insufficient data. Lastly, only around half of the available studies could be included in the dose-response analyses, because many studies only reported dichotomous results. The dose-response analyses therefore need to be interpreted with some caution, and the high versus low analyses are therefore given more weight in the interpretation of the results.

The present study also has several strengths. Unlike the majority of systematic reviews and meta-analyses that only focused on the associations in the general population, we present all available evidence relating to the association of Lp(a) and mortality irrespective of health condition. Additionally, we systematically summarized the available literature presenting the information on the association between Lp(a) and mortality in univariate models or using descriptive/frequency results in our supplementary materials. The linear and non-linear dose-response analyses enabled us to clarify the shape of the dose-response relations and to provide clear insight into the quantitative evaluations of the associations. To identify sources of heterogeneity, we performed various subgroup analysis based on geographical region, follow-up duration, whether studies were conducted in multiple cohorts or a single cohort, adjustment for main confounders, and Lp(a) assessment methods. We transformed the relative risk estimates which were often reported differently by each study to top vs. bottom third of baseline Lp(a) distribution to enable a consistent approach for running the meta-analysis.

According to our findings, we have several suggestions for future studies. Research has indicated that Lp(a) concentrations are mainly determined by the number of KIV2 repeats genetically which inversely correlate with Lp(a) levels [100] and subsequently its function. However, apo(a) isoforms have not been taken into account in the majority of studies. Future studies should take this into consideration. Also, most of the included studies in the analyses were from the US, and some from European countries. Data are lacking for ethnic groups like Africans, which may have higher Lp(a) levels compared to Caucasians [36]. As it was shown in the results of this meta-analysis, regional differences in Lp(a) levels may lead to differences in Lp(a)-associated mortality. Thus, further studies are needed in other geographical regions to provide more clear insight into ethnicity-specific ranges since Lp(a) concentration varies by race/ethnicity [101, 102]. Also, our novel findings of dose-response association between Lp(a) and CVD mortality implicate that therapies which aim at lowering Lp(a) concentration might potentially decrease risk of CVD-death in the general population or in patients with CVD. Thus, this study emphasizes the need for running randomized clinical trials to provide more clear insight on the effects of Lp(a) lowering therapies to prevent all-cause and CVD mortality in populations with different health status. Any further studies should try to report dose-response relationships at more extreme levels of Lp(a) and perform sex-stratified analysis.

In conclusion, our meta-analysis suggests higher risks of all-cause and CVD mortality in both general population (HR: 1.09 for all-cause, HR: 1.18 for CVD mortality) and in patients with CVD (HR: 1.33 for all-cause, HR: 1.25 for CVD mortality) in third versus first tertile of Lp(a) distribution. Also, the linear dose-response analysis showed a higher risk of CVD mortality in both patients with CVD (HR: 1.15) and in the general population (HR: 1.31) per 50 mg/dL increase in Lp(a) concentration. According to 2019 ESC/EAS Guidelines, Lp(a) should be measured at least once in each adult person’s lifetime to identify those who have very high inherited Lp(a) levels > 180 mg/dL, who may have a lifetime risk of atherosclerotic cardiovascular disease which is approximately equivalent to the risk associated with heterozygous familial hypercholesterolemia [7]. Other previous guidelines also recommended Lp(a) measurement in selected cases at high risk, in patients with a family history of premature cardiovascular disease, and for reclassification in subjects with borderline risk [103, 104]. Our findings support these recommendations with regard to the associations of Lp(a) with CVD and all-cause mortality in the general population and in individuals with chronic diseases.

## Electronic supplementary material

Below is the link to the electronic supplementary material.

Supplementary Material 1

## Data Availability

All available data are provided within the manuscript and supplementary files.
