# Supplementary material for: Circulating lipoprotein (a) and all-cause and cause-specific mortality: a systematic review and dose-response meta-analysis
Source: Eur J Epidemiol. 2023 Jan 28;38(5):485–99. doi: 10.1007/s10654-022-00956-4 (PMC10164031; doi:10.1007/s10654-022-00956-4)
Supplement: Supplementary file 1 — Supplementary Material 1 [file 10654_2022_956_MOESM1_ESM.docx]

**Supplements- Circulating lipoprotein (a) and all-cause and cause-specific mortality: A systematic review and dose-response meta-analysis**

**Supplementary Table-1** Search strategy.

| embase | ('lipoprotein A'/de OR (lipoprotein-A OR lipoproteinA OR lp-a OR lp-ai OR lp-aii OR lpa-i OR lpa-ii OR (lpa AND lipoprotein*)):ab,ti) AND ('mortality'/exp OR 'death'/exp OR 'mortality risk'/de OR 'mortality risk score'/de OR 'survival'/exp OR 'survivor'/exp OR (mortalit* OR death* OR fatal* OR surviv*):ab,ti) NOT [conference abstract]/lim NOT ([animals]/lim NOT [humans]/lim) NOT (juvenile/exp NOT adults/exp) NOT ('case report'/de OR case-report:ti) |
| --- | --- |
| Medline ALL Ovid | ("Lipoprotein(a)"/ OR (lipoprotein-A OR lipoproteinA OR lp-a OR lp-ai OR lp-aii OR lpa-i OR lpa-ii OR (lpa AND lipoprotein*)).ab,ti.) AND (exp Mortality/ OR Mortality.fs. OR exp Death/ OR exp Survival/ OR Survivors/ OR (mortalit* OR death* OR fatal* OR surviv*).ab,ti.) NOT (exp animals/ NOT humans/) NOT ((exp infant/ OR exp child/) NOT adult/) NOT (case reports/ OR case-report.ti.) |
| Web of science Core Collection | TS=(((lipoprotein-A OR lipoproteinA OR lp-a OR lp-ai OR lp-aii OR lpa-i OR lpa-ii OR (lpa AND lipoprotein*))) AND ((mortalit* OR death* OR fatal* OR surviv*))) NOT TI=(case-report*) AND DT=(article) |
| Cochrane Central register or trials | ((lipoprotein-A OR lipoproteinA OR lp-a OR lp-ai OR lp-aii OR lpa-i OR lpa-ii OR (lpa AND lipoprotein*)):ab,ti) AND ((mortalit* OR death* OR fatal* OR surviv*):ab,ti) |

**Supplementary Table-2** Characteristics of the included studies in the meta-analysis.

| **Study characteristics** | | | | **Participants’ characteristics** | | | | **Exposure and outcomes** | | | | |
| --- | --- | --- | --- | --- | --- | --- | --- | --- | --- | --- | --- | --- |
|  |  |  |  |  |  |  |  |  | **No. of death** | | |  |
| **Author, year** | **Country** | **Design** | **Follow-up^1^** | **Sex** | **Age^2^** | **Health status** | **No. of Participants** | **Lp(a) assessment method** | **All-cause** | **CVD** | **Non-CVD** | **Adjustments** |
| Perez-Cornago, 2020 [1] | UK | Prospective cohort | 8.7 | Male | 56.53±8.2 | General population | 163,808 | ITA | - | - | 240 | Age (underlying time variable), Townsend deprivation score, ethnicity, lives with a wife or partner, BMI, smoking, PA, alcohol, history of DM. |
| Welsh, 2020 [2] | UK | Prospective cohort | 8.9 | Both | 37-73 | Participants without baseline CVD and not taking a statin | 340,333 | ITA | - | 1,627 | - | Classical risk factors. |
| Welsh, 2020 [2] | UK | Prospective cohort | 8.9 | Both | 37-73 | Participants with baseline CVD and/or taking a statin | 73,391 | ITA | - | 1,620 | - | Classical risk factors. |
| Zhang, 2020 [3] | China | Prospective cohort | 3.02 | Both | 82.86 | AMI | 1,008 | ITA | - | 287 | - | Age, sex, BMI, current smoking, history of HTN/ DM, LDL, heart rate, DBP, left ventricular ejection fraction, eGFR, fasting glucose, hsCRP, revascularization during admission, baseline statin use. |
| Cao, 2020 [4] | China | Prospective cohort | 4.1 | Both | 61.7±16.1 | MI | 3,864 | ITA | - | 241 | - | Age, sex, BMI, family history of CAD, HTN, smoking, history of DM, prerevascularization, Gensini Score, corrected LDL, TG, FBS, hypersensitive, C-reactive protein, baseline statin use. |
| Heinrich, 2020 [5] | Denmark | Prospective cohort | 6.1 | Both | 58.7±8.6 | T2DM with microalbuminuria and no CVD | 198 | Denka Seiken developed Roche second generation | 26 | - | - | Age, sex, smoking, LDL, HbA1c, SBP, creatinine, urinary albumin excretion rate |
| Golledge, 2020 [6] | Australia | Prospective cohort | 2.4 | Both | NM | PAD | 1,472 | Automated latex-enhanced immunoassay | 368 | - | - | Age, sex, smoking, LDL, eGFR, history of DM/ HTN/ CHD, statin prescription |
| Arsenault, 2020 [7] | UK | Prospective cohort | 20 | Both | 45-79 | General population | 18,720 | ITA | 5,686 | 2,412 | - | Age, sex, smoking, BMI, SBP, history of DM, eGFR |
| Liu, 2020 [8] | China | Prospective cohort | 5 | Both | 57.3±10.8 | CAD | 7,562 | ITA | - | 251 | - | Age, sex, smoking, TG, LDL, hs-CRP, HbA1C, history of DM/ HTN, left ventricular ejection fraction, revascularization (percutaneous coronary intervention/coronary artery bypass grafting), baseline statin use |
| Roth, 2020 [9] | Austria | Prospective cohort | 5 | Both | 60.75 | Underwent coronary angiography  for acute coronary syndromes | 1,245 | Isoform-insensitive INA | 195 | 113 | - | Age, sex, BMI, LDL, TG, history of DM/ HTN, presence of  Multi-vessel disease and type of event (ST-segment elevation MI, Non-ST-segment elevation MI or unstable angina) |
| Xu, 2020 [10] | China | Prospective cohort | 6.2 | Both | 61.01±10.06 | Three-Vessel  CAD | 5,130 | ITA | 804 | - | - | Age, sex, LDL, left ventricular ejection fraction, BMI, creatinine clearance, statin treatment, presentation, previous MI, PAD, history of DM/CKD/HTN, SYNTAX Score, treatments |
| Zhang, 2020 [11] | China | Prospective cohort | 3.3 | Both | 58.54±10.47 | T2DM with prior cardiovascular events | 2,284 | ITA | - | 68 | - | Age, sex, smoking, LDL,  FBS, BMI, history of HTN/dyslipidemia, family history of CAD, diseased vessels,  statin and anti-diabetes drugs use |
| Gencer, 2019 [12] | Switzerland | Prospective cohort | 1 | Both | NM | Acute coronary syndromes | 1,711 | INA | 85 | 69 | - | Smoking, LDL, HDL, TG,  BMI, history of HTN/DM. |
| O’Donoghue, 2019 [13]^3^ | 49 countries | Prospective phase  (Clinical trial) | 2.2 | Both | 40-85 | Established atherosclerotic CV disease | 25,096 | Isoform-independent ITA | - | NM | - | Age, sex, smoking, LDL, race, region, prior MI, history of stroke/ PAD/ HTN/ DM, high-intensity statin use, ezetimibe use |
| Langsted, 2019 [14] | Denmark  (2 cohorts) | Prospective cohort | 3-26 | Both | 59.2 | General population | 69,761 | Automated turbidimetric assay, ITA, Isoform insensitive assay | 10,180 | - | - | Age, sex, smoking, BMI, HTN, TG, HDL, CRP, eGFR, ALA, TSH, history of DM, menopausal status and HRT  (women only), Lp(a) corrected total  cholesterol |
| Akinyemiju, 2018 [15] | US | Prospective cohort | 5-9 | Both | ≥ 45 | No active treatment for cancer | 1,764 | INA | - | - | 88 | Age, sex, education, income, site, race, PA, BMI, smoking, alcohol use, comorbidity score |
| Feng, 2017 [16] | China | Prospective cohort | 1.95 | Both | 63.3±10.6 | Undergoing CAG or PCI | 1,684 | Sandwich ELISA | 56 | - | - | Age, sex, LDL, Lp(a) (high), history of HTN/DM, anemia, eGFR, Left ventricular ejection fraction, lesion vessels |
| Bajaj, 2017 [17] | US | Prospective cohort | 7.5 | Both | 58 | Chronic kidney disease | 3,744 | Latex-enhanced ITA | 822 | - | - | Age, sex, race/ethnicity, clinical site, education, tobacco use, SBP, BMI, statin use, TC, LDL, HDL, TG, history of DM/ prior CVD |
| Zewinger, 2017 (original study) [18] | Germany | Prospective cohort | 9.9 | Both | 62.7±10.6 | Established CHD | 3,313 | Photometric assay | 994 | 621 | - | Age, sex, smoking, LDL, DM, SBP, BMI, eGFR, lipid-lowering therapy |
| Zewinger, 2017 (validation study 1)^4^ [18] | Norway | Prospective phase  (Clinical trial) | 10.3 | Both | 61.8±10.4 | Underwent coronary angiography | 4,162 | Particle-enhanced ITA | - | 416 | - | Age, sex, smoking, LDL, DM, SBP, BMI, eGFR, lipid-lowering therapy |
| Zewinger, 2017 (validation study 2)^5^ [18] | Scotland, Ireland, The Netherlands | Prospective phase  (Clinical trial) | 3.2 | Both | 75.6±3.4 | Preexisting vascular disease or an increased risk for vascular disease | 1,860 | Latex agglutination assay | - | 130 | - | Age, sex, smoking, LDL, DM, SBP, BMI, eGFR, lipid-lowering therapy |
| Zewinger, 2017 (validation study 3)^6^ [18] | Germany | Prospective cohort | 4.7 | Both | 61.9±10.1 | Underwent coronary angiography | 2,614 | Particle-enhanced ITA | - | 231 | - | Age, sex, smoking, LDL, DM, SBP, BMI, eGFR, lipid-lowering therapy |
| Waldeyer, 2017 [19] | 5 European countries  (7 cohorts) | Prospective cohort | 9.2 | Both | 42.1-62 | General population | 52,131 | Particle-enhanced ITA | 3,978 | - | - | Age, sex, smoking, LDL, DM, SBP, BMI, eGFR, lipid-lowering therapy |
| Katzke, 2017 [20] | Germany | Case-cohort | 15.6 | Both | 35-66 | General population | 2,739 | NM | - | 381 | 761 | Height, waist, BMI, lifetime alcohol consumption, red meat intake, fiber intake, smoking, socioeconomic  status, PA, history of DM/ HTN, use of lipid-lowering drugs |
| Onat, 2016 [21] | Turkey | Prospective cohort | 5.2 | Both | 59.3±10.65 | General population | 1,274 | INA | 121 | - | - | Age, sex, smoking, HDL, SBP |
| Séguro, 2016 [22] | France | Prospective cohort | 8.6 | Both | 52±10 | General population | 4,930 | NM | 123 | 31 | - | Age, sex, tobacco use, history of HTN/ DM, LDL, statin therapy |
| Zhao, 2016 [23] | US | Prospective cohort | 8.4 | Both | 73.8 | Prior CVD,  Diabetes,  10-year Framingham CVD risk >20% | 3,251 | Monoclonal antibody–based ELISA | 2,928 | - | - | Age, sex, smoking, TG, HDL, SBP, DBP, HTN medication, lipid medication, history of DM, BMI, family history of CHD |
| Nomikos, 2015 [24] | Greece | Prospective cohort | 8.41 | Both | 45±14 | Without any chronic disease | 2,583 | Latex-enhanced ITA | 99 | - | - | Smoking, PA, BMI, history of HTN/DM/ hypercholesterolemia, family history of CVD |
| Patterson, 2015 [25] | UK | Prospective cohort | 15.4 | Male | 45-59 | General population | 1,773 | IRMA | - | 363 | 571 | Age, smoking, DM, SBP, TC, TG, BMI, history of CVD and family history of CHD before 55 years of age |
| Sawabe, 2012 [26] | Japan | Prospective cohort | 12.5 | Both | 55±11.7 | Healthy individuals | 10,413 | ELISA | 830 | 202 | 316 | Age, sex, smoking, BMI, habitual drinking histories |
| Qi, 2012 [27] | US  (2 cohorts) | Prospective cohort | 12-16 | Both | 30-75 | T2DM | 1,686 | Latex-enhanced ITA | - | 156 | - | Age, smoking, BMI, LDL, HDL, TG, HbA1C, alcohol intake, PA, duration of DM, insulin use, aspirin use, cholesterol-lowering medication use, family history of MI, fasting status, history of HTN,  HRT (women only) |
| The Emerging Risk Factors Collaboration, 2009 [28] | Multiple countries  (24-25 studies) | Prospective cohorts/ Nested case-cohorts | 9.3 | Both | 57±8 | General population | 126,634 | - | - | 2,159 | 7,268 | Age, usual levels of SBP, smoking status, history of DM, BMI, TC.  Were stratified where appropriate, by sex and study group |
| Solfrizzi, 2009 [29] | Italy | Prospective cohort | 6.3 | Both | 73.7 ± 5.6 | Elderly individuals | 372 | ELISA | 154 | - | - | Age, sex, number of drinks, geriatric depression scale score, HTN status, smoking, non-HDL, Apo B to Apo A-I ratio, level of fibrinogen, BMI, T2DM, and vascular events at baseline (CAD, stroke, and PAD) |
| Chien, 2008 [30] | China | Prospective cohort | 13.8 | Both | 35-97 | Free of CVD history | 3,484 | Isoform-independent ELISA | 781 | - | - | Age, sex, smoking, current alcohol drinking, HDL, LDL, BMI, marital status, education, occupation, PA, family history of CHD, history of HTN/ DM |
| D’Angelo, 2006 [31] | Italy | Prospective cohort | 6.3 | Both | 65-87 | With or without history of vascular disease | 559 | ELISA | 107 | 33 | - | Age, sex |
| Hernandez, 2005 [32] | Spain | Prospective cohort | 10 | Both | 59.1±11 | T2DM | 100 | ELISA | - | 23 | - | Age, sex, BMI, smoking, HbA1c, LDL, HDL, TG, creatinine, albumin excretion rate, and the presence of HTN, macroangiopathy, retinopathy |
| Ariyo, 2003 [33] | US | Prospective cohort | 7.4 | Female | 72.2±5.4 | Without vascular disease | 2,375 | Monoclonal antibody–based ELISA | NM | - | - | Age, sex, TC, LDL, TG, smoking, history of DM/HTN, BMI, carotid-wall thickness, estrogen use or nonuse, other traditional risk factors |
| Ariyo, 2003 [33] | US | Prospective cohort | 7.4 | Male | 73.2±5.8 | Without vascular disease | 1,597 | Monoclonal antibody–based ELISA | NM | - | - | Age, sex, TC, LDL, TG, BMI, carotid-wall thickness, smoking, history of DM/ HTN, other traditional risk factors |
| Ahlbeck glader, 2002 [34] | Sweden | Prospective cohort | 6.7 | Both | 59.4±8.1 | Stable effort angina | 1,216 | In-house ELISA | 200 | 152 | - | Age, sex, smoking, LDL, TC, HDL, TG, fibrinogen, BMI, Myocardial coronary obstruction scores, Left ventricular motion scores, previous AMI, HTN, history of stroke/claudication/DM, AT III and SR |
| Iliescu, 2002 [35] | Canada | Prospective cohort | 2 | Both | 58.1±15.9 | Peritoneal dialysis | 54 | ELISA | 24 | - | - | Age |
| Zairis, 2002 [36] | Greece, New York | Prospective cohort | 1.84 | Both | 59.3±10 | Stable or unstable coronary syndromes (after successful coronary stenting) | 483 | Immunoassay method | - | 21 | - | Other univariate predictors of the event |
| Lundstam, 2002 [37] | Sweden | Prospective cohort | 11.7 | Both | 17-78 | CHD patients who underwent coronary angiography | 964 | ELISA | 363 | - | - | Age, sex |
| Cheng, 2001 [38] | China | Prospective cohort | 3.6 | Both | 37-87 | Lower limb arterial occlusive disease (intermittent claudication, critical ischemia) | 441 | ITA | 144 | - | - | For the effects of all other confounding variables |
| Gerdes, 2000 [39]^7^ | Denmark | Prospective phase  (Clinical trial) | 5.5 | Both | 35-70 | MI survivors | 966 | IRMA | 54 | 38 | - | Age, sex, smoking, HDL, LDL, TG, Apo A-I, Apo B, Lp(a), ꞓ4-carrier status, nationality, angina pectoris, history of DM/ HTN/claudication |
| Shlipak, 2000 [40]^8^ | US | Prospective phase  (Clinical trial) | 4.1 | Female | 66.7 | Postmenopausal women with CAD | 1,383 | ELISA | - | 59 | - | Race/ethnicity, history of DM, WHR, tobacco use, HDL, LDL, TG, and use of lipid-lowering agents, aspirin, and calcium channel blockers |
| Koda, 1999 [41] | Japan | Prospective cohort | 2.3 | Both | 56.6±14.1 | Hemodialysis | 390 | Latex-enhanced ITA | 49 | 18 | - | Age, history of DM, albumin |
| Ohashi, 1999 [42] | Japan | Prospective cohort | 5 | Both | 26-73 | Hemodialysis | 268 | ELISA |  | 70 | - | NM |
| Stubbs, 1998 [43] | UK | Prospective cohort | 3 | Both | 63 | MI | 266 | ELISA | - | 57 | - | Age, previous MI, infarct size, HTN |
| Stubbs, 1998 [43] | UK | Prospective cohort | 3 | Both | 60.8 | Unstable angina | 197 | ELISA | - | 31 | - | Conventional risk factors, accelerated angina |

^1^ Follow-up is presented as median, or mean, or min and max of years.  ^2^Age is reported as Mean ± SD, Median (range), or min-max.^3^ Results of placebo group of FOURIER Trial (prospective phase) are presented. ^4^The prospective phase of the clinical trial study (The Western Norway B Vitamin Interventional Trial and The Bergen Coronary Angiography Cohort (WENBIT/BECAC cohort)) was included. ^5^The prospective phase of the clinical trial study (The PROspective Study of Pravastatin in the Elderly at Risk (PROSPER)) was included. ^6^ The ATHEROGENE study which is a prospective study on patients underwent coronary angiography was reported. ^7^ Results of placebo group of Scandinavian Simvastatin Survival Study (prospective phase) were included. ^8^ The results of the prospective phase of the clinical trial for the placebo group are reported. NM: Not mentioned; AMI: acute myocardial infarction; Apo: Apolipoprotein; ALA: Alanine aminotransferase; BMI: Body mass index; CAD: Coronary artery diseases; CVD: Cardiovascular diseases; CAG: Coronary angiography; CHD: Coronary heart diseases; CKD: chronic kidney disease; DM: Diabetes mellitus; ELISA: Enzyme-linked immunosorbent assay; eGFR: estimated glomerular filtration rate; FBS: fasting blood sugar; HTN; Hypertension; HDL: High-density lipoprotein cholesterol; HbA1c: Hemoglobin A1C; HRT: Hormone replacement therapy; ITA: Immunoturbidimetric assay; INA: Immunonephelometric assay; IRMA: Immunoradiometric assay; Lp(a): Lipoprotein (a); LDL: Low-density lipoprotein cholesterol; MI: myocardial infarction; PA: Physical activity; PAD: Peripheral artery disease; PCI: Percutaneous coronary intervention; SBP: Systolic blood pressure; TG: Triglyceride; TSH: thyroid stimulating hormone; T2DM: Type 2 diabetes; WHR: waist-to-hip ratio.

**Supplementary Table-3** Quality of the included studies in the systematic review using the Newcastle Ottawa Scale.

| **Author, year** | **Selection** | | | | **Comparability** | **Outcome/ Exposure** | | | **Total Score** |
| --- | --- | --- | --- | --- | --- | --- | --- | --- | --- |
|  | **1** | **2** | **3** | **4** | **1** | **1** | **2** | **3** | **9** |
| Perez-Cornago, 2020 [1] | * | * | * | * | ** | * | * | * | 9 |
| Welsh, 2020 [2] | * | * | * | * | ** | * | * | * | 9 |
| Zhang, 2020 [3] | - | * | * | - | ** | * | - | * | 6 |
| Cao, 2020 [4] | - | * | * | - | ** | * | - | * | 6 |
| Heinrich, 2020 [5] | - | * | * | - | ** | - | * | * | 6 |
| Golledge, 2020 [6] | - | * | * | - | ** | * | - | * | 6 |
| Arsenault, 2020 [7] | * | * | * | * | ** | * | * | * | 9 |
| Liu, 2020 [8] | - | * | * | - | ** | - | * | * | 6 |
| Roth, 2020 [9] | - | * | * | - | ** | * | * | * | 7 |
| XU, 2020 [10] | - | * | * | - | ** | * | * | * | 7 |
| Zhang, 2020 [11] | - | * | * | - | ** | * | - | * | 6 |
| Gencer, 2019 [12] | - | * | * | - | ** | * | - | * | 6 |
| O’Donoghue, 2019 [13] | - | * | * | - | ** | * | - | * | 6 |
| Langsted, 2019 [14] | * | * | * | * | ** | * | * | * | 9 |
| Akinyemiju, 2018 [15] | * | * | * | * | ** | * | * | * | 9 |
| Feng, 2017 [16] | - | * | * | - | ** | - | - | * | 5 |
| Bajaj, 2017 [17] | - | * | * | - | ** | * | * | * | 7 |
| Zewinger, 2017 (original study) [18] | - | * | * | - | ** | * | * | * | 7 |
| Zewinger, 2017 (validation study 1)^1^ [18] | - | * | * | - | ** | * | * | * | 7 |
| Zewinger, 2017 (validation study 2)^2^ [18] | - | * | * | - | ** | * | - | * | 6 |
| Zewinger, 2017 (validation study 3)^3^ [18] | - | * | * | - | ** | * | - | * | 6 |
| Waldeyer , 2017 [19] | * | * | * | * | ** | - | * | * | 8 |
| Katzke, 2017 [20] | * | * | - | * | ** | * | * | * | 8 |
| Onat, 2016 [21] | * | * | * | * | ** | * | * | * | 9 |
| Séguro, 2016 [22] | * | * | - | * | ** | * | * | * | 8 |
| Zhao, 2016 [23] | - | * | * | - | ** | * | * | * | 7 |
| Nomikos, 2015 [24] | * | * | * | * | ** | - | * | * | 8 |
| Patterson, 2015 [25] | * | * | * | * | ** | * | * | * | 9 |
| Sawabe, 2012 [26] | * | * | * | * | ** | * | * | * | 9 |
| Qi, 2012 [27] | - | * | * | - | ** | * | * | * | 7 |
| Solfrizzi, 2009 [29] | * | * | * | * | ** | * | * | * | 9 |
| The Emerging Risk Factors Collaboration, 2009 [28] | * | * | * | * | ** | * | * | * | 9 |
| Chien, 2008 [30] | * | * | * | * | ** | * | * | * | 9 |
| D’Angelo, 2006 [31] | * | * | * | * | * | * | * | * | 8 |
| Hernandez, 2005 [32] | - | * | * | - | ** | - | * | * | 6 |
| Ariyo, 2003 [33] | * | * | * | * | ** | * | * | * | 9 |
| Ahlbeck glader, 2002 [34] | - | * | * | - | ** | * | * | * | 7 |
| Iliescu, 2002 [35] | - | * | * | - | * | * | - | * | 5 |
| Zairis, 2002 [36] | - | * | * | - | ** | * | * | * | 7 |
| Lundstam, 2002 [37] | - | * | * | - | * | * | * | * | 6 |
| Cheng, 2001 [38] | - | * | * | - | ** | * | - | * | 6 |
| Gerdes, 2000 [39] | - | * | * | - | ** | - | * | * | 6 |
| Shlipak, 2000 [40] | - | * | * | - | ** | * | - | * | 6 |
| Koda,1999 [41] | - | * | * | - | * | * | - | * | 5 |
| Ohashi,1999 [42] | - | * | * | - | * | - | * | * | 5 |
| Stubbs, 1998 [43] | - | * | * | - | ** | * | - | * | 5 |

**Supplementary Table-4** Summary of the included studies in the systematic review, reporting the death number/percent in low/high level of Lp(a).

| **Study characteristics** | | | | **Participants’ characteristics** | | | | | **Exposure and results** | | |
| --- | --- | --- | --- | --- | --- | --- | --- | --- | --- | --- | --- |
| **Author, year** | **Country** | **Design** | **Follow-up^1^** | **Sex** | **Age^2^** | | **Health status** | **No. of participants/ low Lp(a) group** | **Lp(a) assessment method** | **Lp(a) classification** | **Summary of results** |
|  |  |  |  |  | **Low Lp(a)** | **High Lp(a)** |  |  |  |  |  |
| Bigazzi, 2021 [44] | Italy | Prospective cohort | 2 | Both | 66.3 ± 12.3 | 66.3 ± 12.5 | Coronary ischemia | 2374/ 1576 | Rate nephelometry | ≤30, >30–50, ≥50 mg/dL | 21 deaths were observed in the lowest and 2 in the highest levels of Lp(a). Among population with Lp(a) concentration between 30-50 mg/dL, 7 deaths were reported. |
| Hishikari, 2020 [45] | Japan | Prospective cohort | 2 | Both | 70.6 ± 8.3 | 71.2 ± 9.9 | Hemodialysis | 410/ 297 | Latex agglutination immunoassay | <40, ≥40 mg/dL | No significant differences were observed in cardiac death between two levels of Lp(a)  (Low Lp(a): 18 (6.1%) vs. high Lp(a): 13 (11.5%), p = 0.092) |
| Matsushita, 2020 [46] | Japan | Prospective phase  (Clinical trial) | 0.83 | Both | 63±10 | 67±11 | Acute coronary syndrome | 76/ 49 | Latex agglutination immunoassays | ≤ 20, > 20 mg/dL | No significant differences were observed between two levels of Lp(a) (Low Lp(a): 6 vs. high Lp(a): 0, p = 0.55). |
| Dewa Gde Dwi Sumarjaya, 2020 [47] | Indonesia | Prospective cohort | NM | Both | 56.45±9.48 | 61.69±10.47 | Hospitalized-acute MI | 66/ 31 | ITA | < 10.25, ≥ 10.25 mg/dL | 3.2 % (Low Lp(a) group) and 11.4 % (High Lp(a) group) CVD mortality were observed. |
| Xu, 2020 [48] | China | Prospective cohort | 2.4 | Both | 57.6±10.3 | 58.5±10 | Patients underwent PCI | 6714/ 2238 | latex turbidimetric method | ≤ 10.59 or 10.59-30.55 or ≥ 30.55 mg/dL | No significant differences were observed in different levels of Lp(a) for cardiovascular death (tertile 1: 7 (0.3 %), tertile 2: 6 (0.3 %), tertile 3: 8 (0.4 %), and all-cause death tertile 1: 26 (1.2 %), tertile 2: 15 (0.7 %), tertile 3: 15 (0.7 %)). |
| Shitara, 2019 [49] | Japan | Prospective cohort | 5.1 | Both | 65.2±12 | 65.3±11.5 | Coronary artery disease and Left  ventricular systolic dysfunction | 369/ 184 | Latex agglutination immunoassays | < 21.6 or ≥ 21.6 mg/dL | 36 (23.8 5) and 44 (29.1 %) all-cause death and 13 (8.6 %) 22 (14.6 %) cardiovascular death were observed in low Lp(a) and high Lp(a) groups, respectively. |
| Sanchez Munoz-Torrero, 2018 [50] | Spain | Prospective cohort | 3 | Both | 67±11 | 66±11 | Stable outpatients with symptomatic artery  disease | 1503/ 814 | Kinetic nephelometric | < 30 or  30-50 or ≥ 50 mg/dL | No significant differences were observed in different levels of Lp(a) for cardiovascular death (Lp < 30: 20 (2.5 %), Lp: 30-50: 4 (1.3 %), Lp ≥ 50: 13 (3.5 %), non-cardiovascular death (Lp < 30: 24 (2.9 %), Lp = 30-50: 8 (2.5 %), Lp ≥ 50: 16 (4.3 %) and all-cause death (Lp < 30: 44 (5.4 %), Lp: 30-50: 12 (3.8 %), Lp ≥ 50: 29 (7.8 %)) |
| Fogacci, 2017 [51] | Italy | Prospective cohort | 25 | Both | NM | NM | Without history of CHD | 1215 | ELISA | Stratified into quartiles | Subjects at high and intermediate CV risk aged 56–69 years (regardless of gender) and women aged 40–55 years with a low CV risk profile who had lower Lp(a) levels showed a significant benefit on CV mortality (p < 0.05). |
| Xie, 2017 [52] | China | Prospective cohort | 2.6 | Both | 57±10 | 60±8 | Patients with non-obstructive CAD | 451/ 150 | Latex agglutination immunoassays | 0.2-10.6 or 10.9-23.4 or 23.8-124 mg/dL | Significant differences were observed across different levels of Lp(a) and cardiovascular death (tertile 1: 2 (1%), tertile 2: 4 (3%), and tertile 3: 9 (6 %)). |
| Konishi, 2016 [53] | Japan | Prospective cohort | 4.7 | Both | 70.1±9.2 | 69.9±9.4 | CKD | 904/ 454 | Latex agglutination immunoassays | According to median levels of Lp(a) | The difference in cardiac death was significant between two levels of Lp(a) (Low Lp(a): 38 (8.4%) vs. high Lp(a): 67 (14.8%), p < 0.01).  No considerable differences were observed in Non-cardiac death (Low Lp(a): 52 (11.6%) vs. high Lp(a): 54 (11.9%), p = 0.88) |
| Mitsuda, 2016 [54] | Japan | Prospective cohort | 3 | Both | 64±12.4 | 66±12.8 | STEMI patients after primary percutaneous coronary intervention | 176/ 88 | Latex agglutination immunoassays | < 16.5 or >16.5 mg/dL | 0 (0%) death in low Lp(a) group and 1 (1.1%) death in high Lp(a) group were observed. |
| Konishi, 2015 [55] | Japan | Prospective cohort | 4.7 | Both | 67.8±10.4 | 67±10.7 | CAD | 411/ 292 | Latex agglutination immunoassays | < 30 or ≥ 30 mg/dL | 34 deaths in low Lp(a) group and 19 deaths in high Lp(a) group were observed. |
| Cai, 2013 [56] | China | Prospective phase  (Clinical trial) | 1 | Both | 62.2±10.3 | 62.3±11.1 | CAD, eligible for stent placement | 832/ 552 | Sandwich ELISA | < 30 or > 30  mg/dL | In both subgroups of baseline LDL-C ( ≥ 1.8 and <18 mmol/L), no significant differences were observed in CVD mortality between two groups of Lp(a).  (LDL < 18 mmol/L, low Lp(a): 6 (2.1%) vs. high Lp(a): 2 (2.2%)  LDL ≥ 1.8, low Lp(a): 15 (5.7%) vs. high Lp(a): 10 (5.3%)) |
| Zhou, 2012 [57] | China | Prospective cohort | 2.5 | Both | NM | NM | Acute coronary syndrome | 713/ 178 | Immune turbidimetric assay | < 9 or 9-< 15.2 or 15.2 < 31.3 or ≥ 31.3 mg/dL | Number of cardiovascular death events were 1, 4, 5, 4 for quartiles 1 to 4, respectively. |
| Ikenaga, 2011 [58] | Japan | Prospective cohort | 5 | Both | 63±12 | 64±11 | Acute MI | 410/ 315 | Turbidimetric immunoassay system | ≤ 40 or > 40 mg/dL | The difference in cardiac death was significant between two levels of Lp(a)  (Low Lp(a): 5 (1.6%) vs. high Lp(a): 5 (5.3%), p = 0.06) |
| Boras, 2010 [59] | Croatia | Prospective cohort | 4 | Both | 63 (45-69) | 64 (46-70) | T2DM | 146/ 60 | Automated ITA | ≤ 30 or > 30  mg/dL | There were no significant differences in CVD or stroke death between two Lp(a) groups. |
| Nicholls, 2010 [60] | US | Prospective cohort | 3 | Both | 63.9±10.9 | 63±10.7 | Patients underwent coronary angiography | 2769/ 1720 | Automated latex enhanced  immunoassay | < 30 or ≥ 30 mg/dL | The rate of death did not significantly differ between patients in low Lp(a) (10.5 %) and high Lp(a) (12.2 %) levels (p = 0.20) |
| Igarashi, 2003 [61] | Japan | Prospective cohort | 2.9 | Both | 67±7 | 64±8 | Acute MI | 127/ 96 | Latex-enhanced turbidimetric immunoassay | < 47 or ≥ 47  mg/dL | 2.1 % cardiac death were accrued in participants with low Lp(a) level although no cardiac mortality was observed in the other group (p > 0.05). Two and one fatal MI were observed in high and low Lp(a) group (p > 0.05). |
| Longenecker, 2002 [62] | US | Prospective cohort | 2.8 | Both | NM | NM | Dialysis patients | 864/ 433 | ELISA | < 53 or ≥ 53 nmol/L | 187 and 158 deaths were occurred in low and high Lp(a) groups, respectively. |
| Cristóbal, 1999 [63] | Spain | Prospective cohort | > 3 | NM | 43 | 48 | Cardiac transplant recipients | 166/ 108 | ELISA | < 30 or > 30  mg/dL | Three-year global mortality was not significantly different between Lp(a) groups. Significant CVD mortality (Low Lp(a): 12% vs. high Lp(a): 3%) and sudden death (Low Lp(a): 4% vs. high Lp(a): 0%) were observed. |
| Skinner, 1997 [64] | UK | Prospective cohort | 5 | Both | NM | NM | patients underwent first time CABG for stable angina | 353/ 116 | Enzyme-linked immunosorbent assay | < 123 or 123-331 or 331-1411 mg/l | Late cardiac death was 5, 8, 2 events in tertiles 1 to 3 of Lp(a) levels. Thus, serum Lp(a) concentration did not predict late cardiac death. |
| Goldwasser, 1993 [65] | US | Prospective cohort | 1 | Both | 60±16 | 61±13 | Hemodialysis | 125/ 84 | ITA | < 57 or ~ 57  mg/dL | Lower Lp(a) level was associated with a survival of 78% compared with 83.7% for Lp(a) ~ 57 mg/dL  (p = 0.23 by log-rank test). |

^1^ Follow-up is presented as median, mean, min and max of years.  ^2^ Age is reported as Mean ± SD, Median (range), or min and max. NM: Not mentioned; T2DM: Type 2 diabetes mellitus; CAD: Coronary artery disease; MI: Myocardial infarction; CHD: Coronary heart diseases; CKD: Chronic kidney disease; CABG: Coronary artery bypass graft surgery; PCI: Percutaneous coronary intervention; ITA: Immunoturbidimetric assay; ELISA: Enzyme-linked immunosorbent assay.

**Supplementary Table-5** Summary of the included studies in the systematic review, reporting level of Lp(a) in survivors/non-survivors.

| **Study characteristics** | | | | **Participants’ characteristics** | | | | | **Exposure and results** | | | |
| --- | --- | --- | --- | --- | --- | --- | --- | --- | --- | --- | --- | --- |
| **Author, year** | **Country** | **Design** | **Follow-up** | **Gender** | **Age^1^** | | **Health status** | **No. of participants/ Survivors** | **Lp(a) assessment method** | **Lp(a) level** | | **Summary of results** |
|  |  |  |  |  | **Survivor** | **Non-survivor** |  |  |  | **Survivors** | **Non- survivors** |  |
| Schwaiger, 2006 [66] | Austria | Prospective cohort | 13 | Both | 43 ± 10.9 | 59 ± 10.7 | Hemodialysis  patients | 165/ 70 | ELISA | 20.6 ± 27.3  mg/dL | 30.1 ± 30.7  mg/dL | Significant difference was observed  (p = 0.025) |
| Hocher, 2003 [67] | Germany | Prospective cohort | 2 | Both | 63.5 ± 5.8 | 70.4 ± 9.8 | Hemodialysis  patients | 245/ 172 | Standardized autoanalyzer methods | 29.5 ± 33.2 mg/dL | 29.3 ± 32.5 mg/dL | No significant difference was observed. |
| Zimmermann, 1999 [68] | Germany | Prospective cohort | 2 | Both | NM | NM | Hemodialysis  patients | 280/ 208 | Nephelometric immunoassay | 9.2 mg/dL | 13.6 mg/dL | After 4 years of follow-up, 42 cardiovascular deaths and 72 all-cause deaths were reported |
| Koch, 1997 [69] | Germany | Prospective cohort | 9 | Both | 53 ± 15 | 61 ± 12 | Diabetic patients with ESRD | 412/ 232 | Electroimmuno-diffusion | 22.6 ± 22  mg/dL | 24.1 ± 21.4  mg/dL | No significant difference was observed  (p = 0.9) |
| Cressman, 1994 [70] ^2^ | US | Prospective cohort | 4 | Both | 57 ± 12 | 64 ± 11 | Patients with ESRD | 129/ 62 | Double-antibody radioimmunoassay | 3.33 ± 1.08  (log Lp(a), mg/dL) | 3.72 ±0.9  (log Lp(a), mg/dL) | Significant difference was observed  (p < 0.005) |
| Cressman, 1992 [71] ^2^ | US | Prospective cohort | 4 | Both | 58 ± 14 | 58 ± 14 | Hemodialysis Patients | 129/ 60 | Double-antibody radioimmunoassay | 37.7 mg/dL | 46.7 mg/dL | After 4 years of follow-up, 11 non-cardiovascular deaths and 13 uncertain deaths were reported |
| Haffner, 1992 [72] | US | Nested case-control | 4 | Both | NM | NM | Patients with diabetes | 70/ 35 | Monoclonal anti-Lp(a) antibody | Older-onset:  12.7 ± 3.7  Younger-onset:  18.5 ± 6.6  (mg/dL) | Older-onset:  15.4 ± 3.9  Younger-onset:  16.8 ± 6.6  (mg/dL) | No significant difference was observed  (older-onset: p = 0.60,  younger-onset: p = 0.86) |

^1^Age is reported as Mean or Median of years. ^2^ These studies are derived from a same population but reported different study outcomes. NM: Not mentioned; NS: Non-significant; ESRD: End-stage renal disease; ELISA: Enzyme-linked immunosorbent assay.


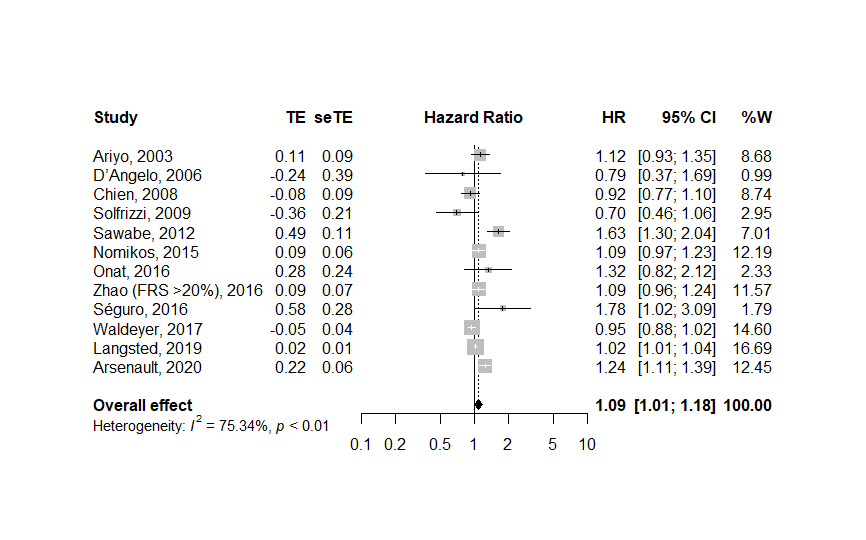


**Supplementary Figure-1** Summary hazard ratios of all-cause mortality for top versus bottom third of Lp(a) levels in general population.


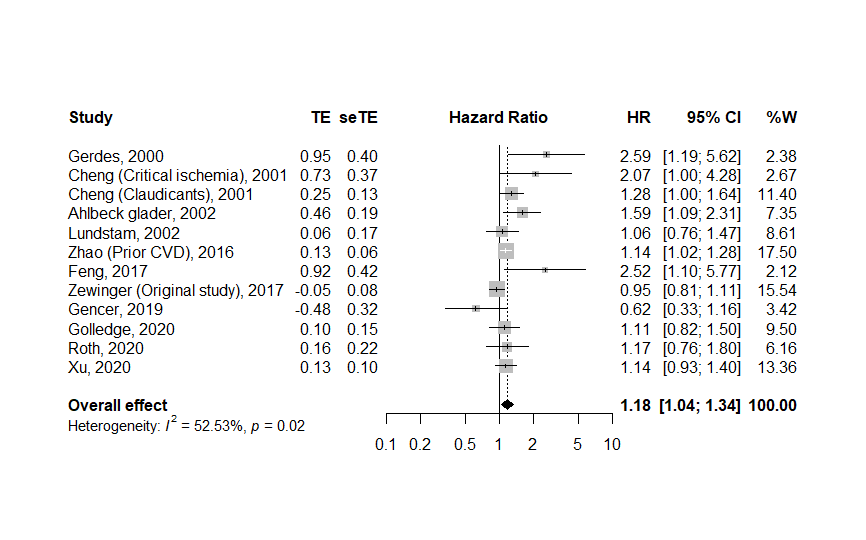


**Supplementary Figure-2** Summary hazard ratios of all-cause mortality for top versus bottom third of Lp(a) levels in patients with CVD.


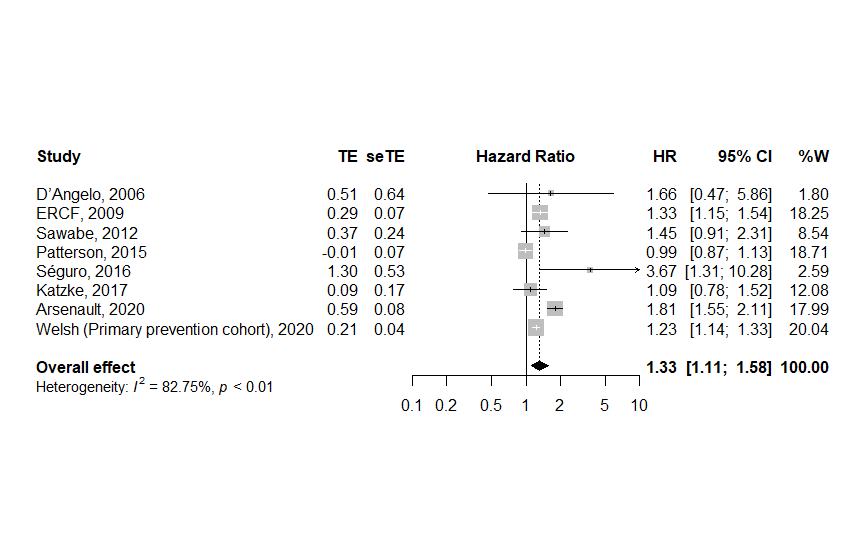


**Supplementary Figure-3** Summary hazard ratios of CVD-death for top versus bottom third of Lp(a) levels in general population.


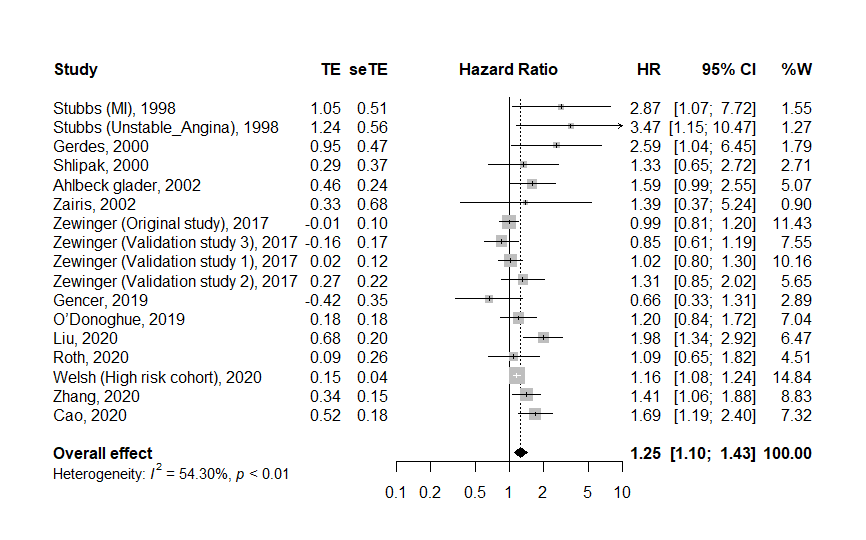


**Supplementary Figure-4** Summary hazard ratios of CVD-death for top versus bottom third of Lp(a) levels in patients with CVD.


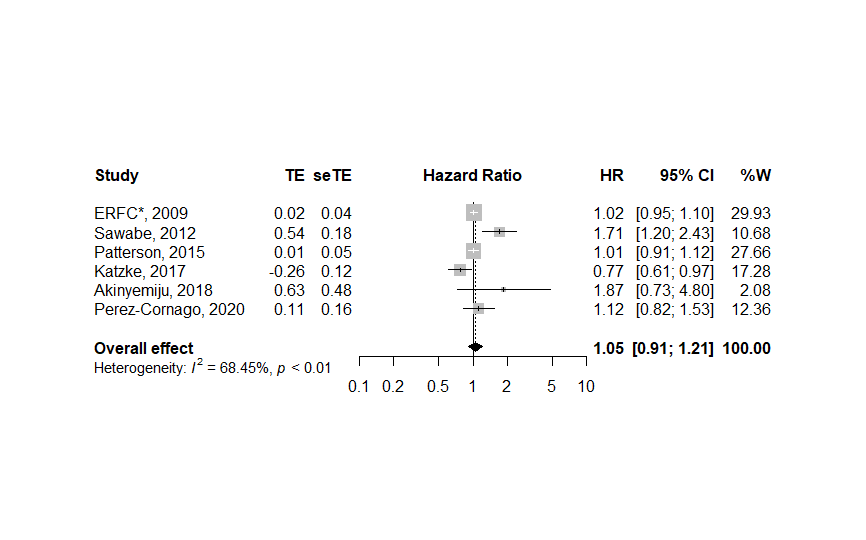


**Supplementary Figure-5** Summary hazard ratios of non-CVD-death for top versus bottom third of Lp(a) levels in general population. ^*^ERFC: The emerging risk factor collaboration.


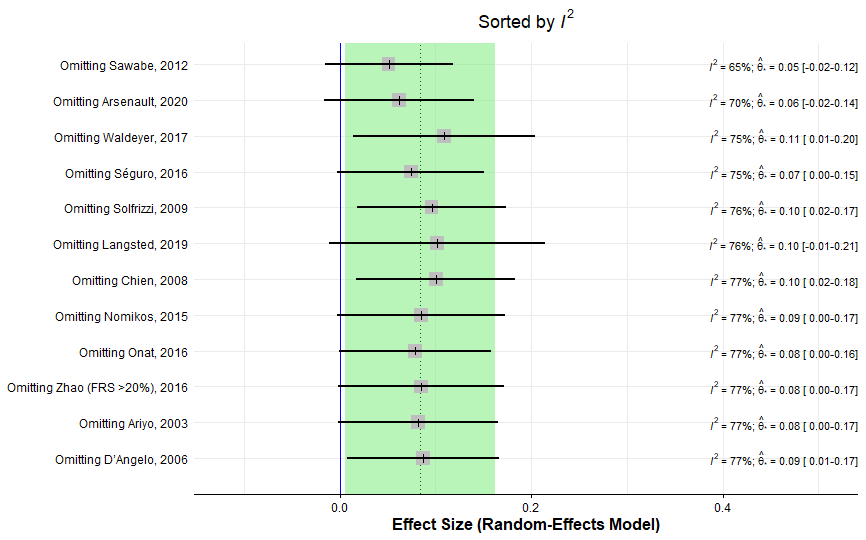


A


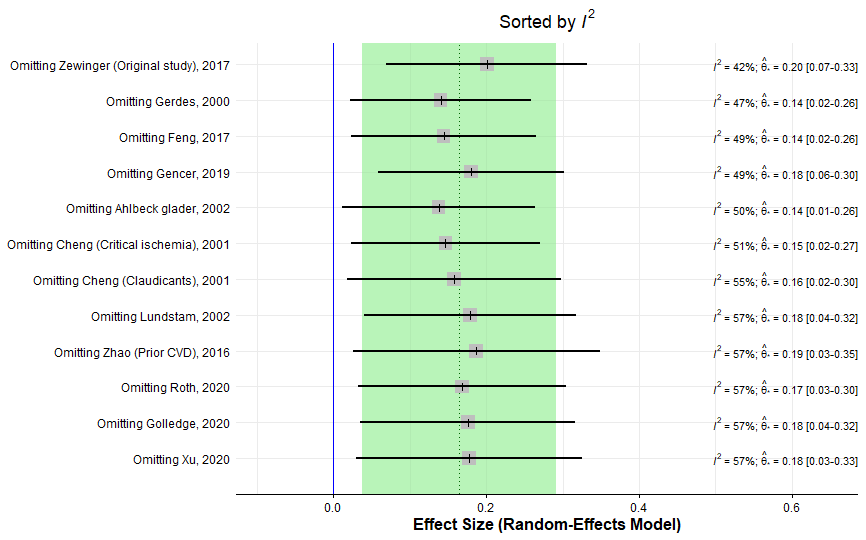


B


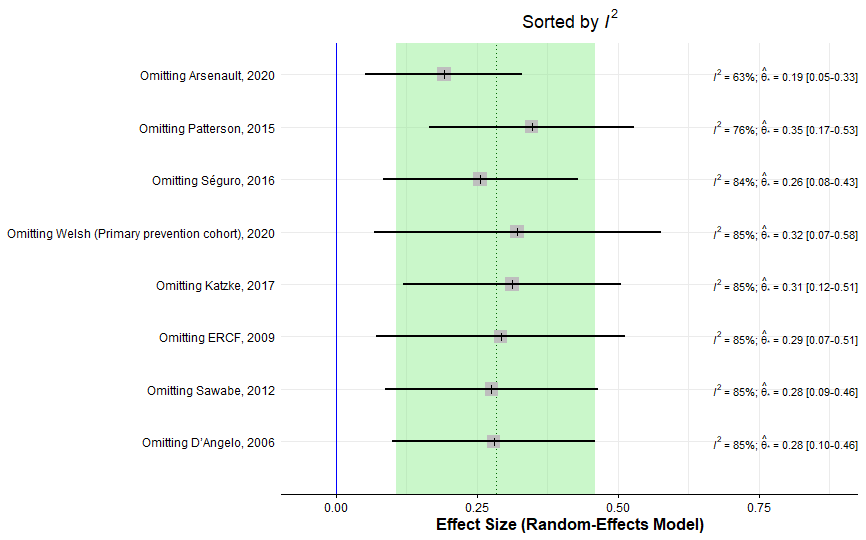


C


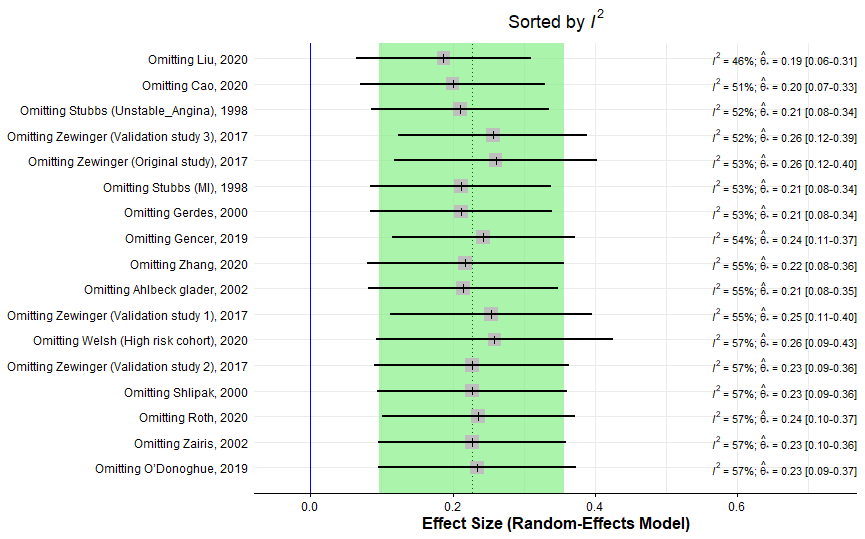


D


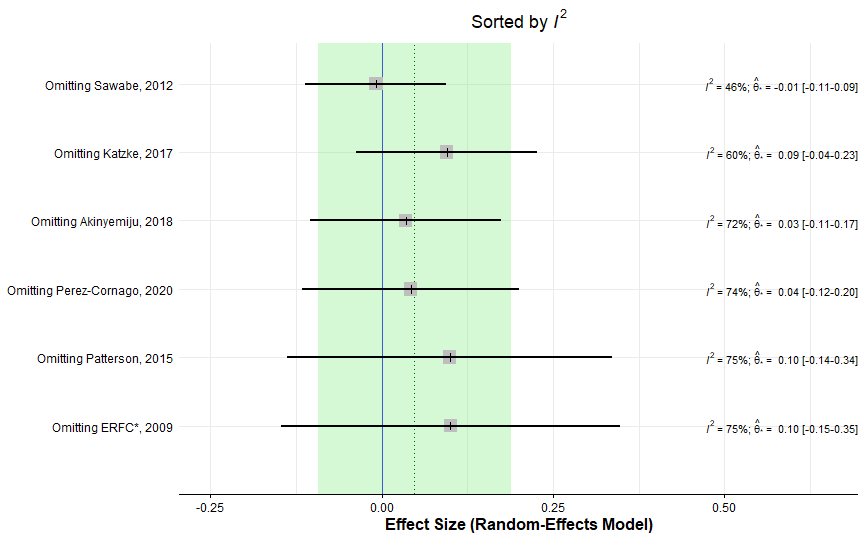


E

**Supplementary Figure-6** Leave-one-out forest plots of the included studies in meta-analysis of **A**) All-cause mortality in general population, **B**) All-cause mortality in patients with CVD, **C**) CVD- death in general population, **D**) CVD-death in patients with CVD, **E**) Non-CVD-death in general population. Effects sizes are sorted by I^2^.


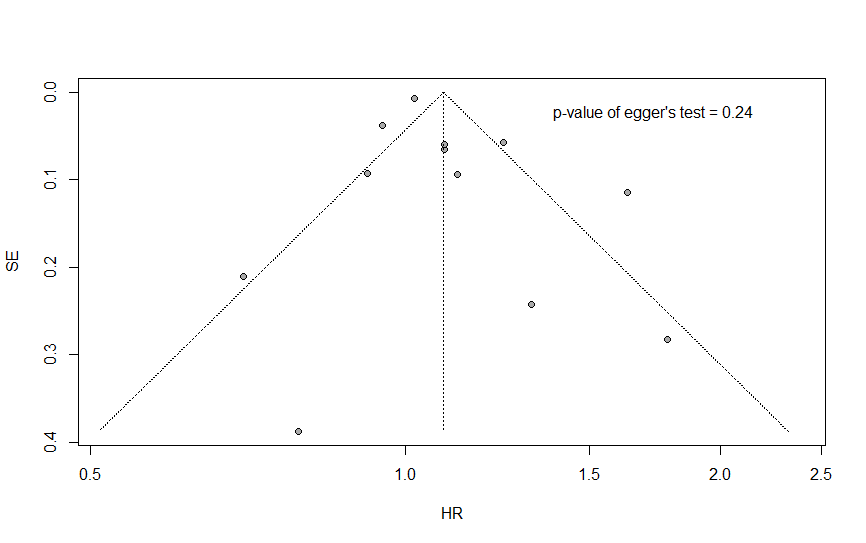


A


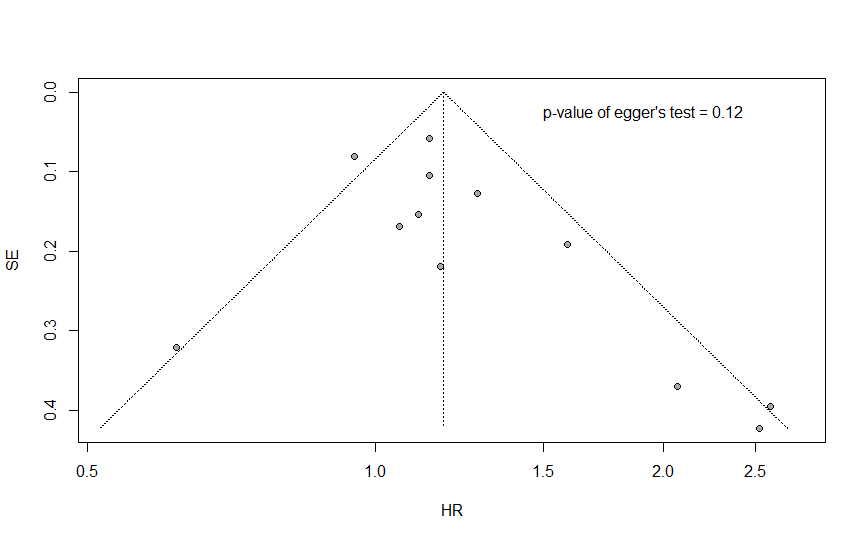


B


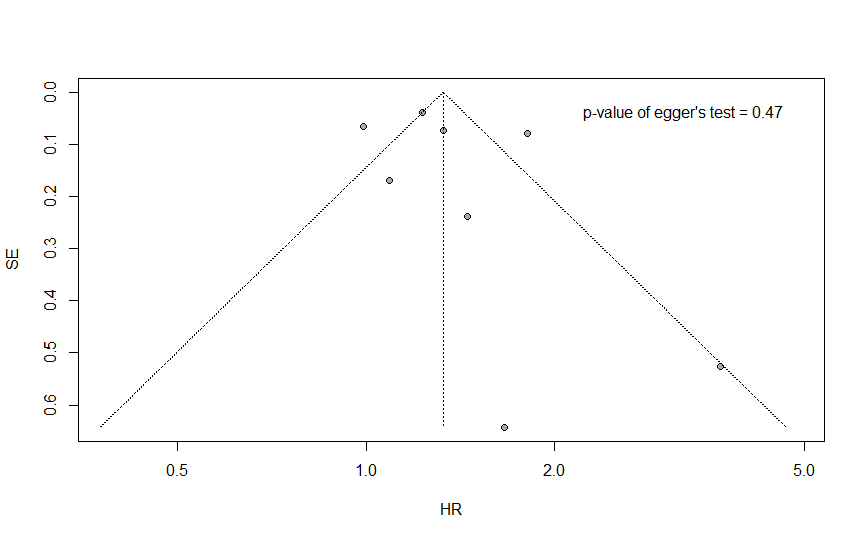


C


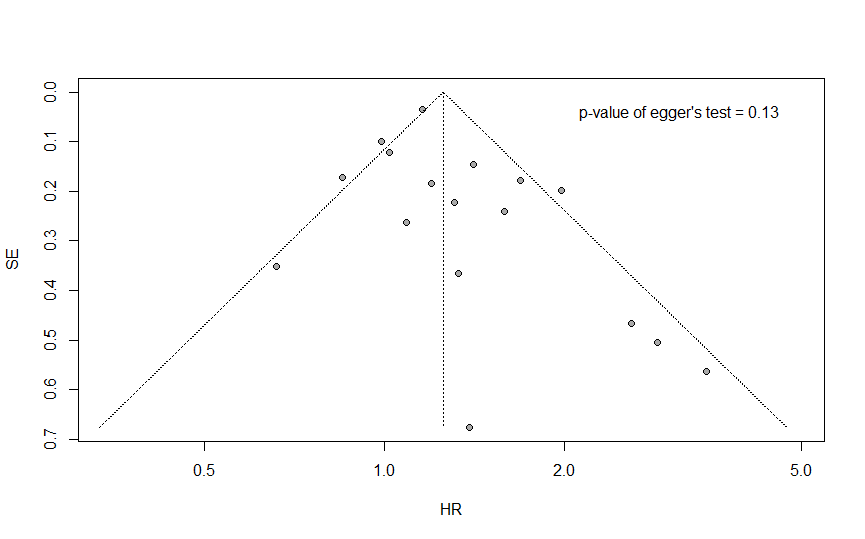


D


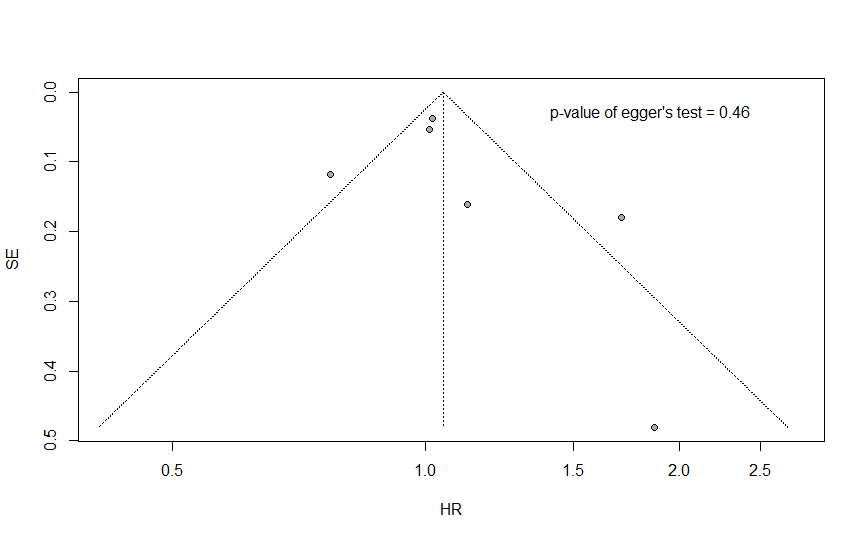


E

**Supplementary Figure-7** Funnel plots of the hazard ratios of the included studies in meta-analysis of **A**) All-cause mortality in general population, **B**) All-cause mortality in patients with CVD, **C**) CVD- death in general population, **D**) CVD-death in patients with CVD, **E**) Non-CVD-death in general population. X and Y- axes represent the HR and SE, respectively.

A

B

C

D

E

**Supplementary Figure-8** Non-linear dose response analysis. **A**) All-cause mortality in general population, **B**) All-cause mortality in patients with CVD, **C**) CVD- death in general population, **D**) CVD-death in patients with CVD, **E**) Non-CVD-death in general population. X and Y- axes represent the Lp(a) concentration in mg/dl and HR, respectively.

**Supplementary Table-6** Table of HRs from nonlinear dose-response analyses.

|  | **All-cause mortality** | | **CVD-death** | | **Non-CVD-death** |
| --- | --- | --- | --- | --- | --- |
| **Lp(a)** | **General population** | **CVD patients** | **General population** | **CVD patients** | **General population** |
| mg/dl | HR (95% CI) | HR (95% CI) | HR (95% CI) | HR (95% CI) | HR (95% CI) |
| 0 | 1.00 | 1.00 (3.25 mg/dl) | 1.00 (1.45 mg/dl) | 1.00 (3.25 mg/dl) | 1.00 (1.45 mg/dl) |
| 10 | 1.00 (0.91-1.10) | 1.00 (0.94-1.06) | 1.02 (0.83-1.24) | 1.07 (0.99-1.15) | 0.95 (0.87-1.04) |
| 20 | 1.01 (0.84-1.20) | 0.99 (0.87-1.12) | 1.05 (0.77-1.44) | 1.16 (0.98-1.36) | 0.90 (0.76-1.08) |
| 30 | 1.02 (0.82-1.28) | 0.98 (0.83-1.15) | 1.13 (0.80-1.60) | 1.21 (0.98-1.51) | 0.87 (0.69-1.11) |
| 40 | 1.05 (0.81-1.35) | 0.96 (0.81-1.14) | 1.26 (0.69-2.28) | 1.24 (0.97-1.58) | 0.86 (0.66-1.12) |
| 50 | 1.07 (0.81-1.42) | 0.94 (0.78-1.13) | 1.42 (0.46-4.38) | 1.24 (0.97-1.59) | 0.87 (0.68-1.11) |
| 60 | 1.10 (0.82-1.49) | 0.92 (0.72-1.17) | 1.65 (0.27-10.11) | 1.23 (0.96-1.57) | 0.90 (0.73-1.11) |
| 70 | 1.14 (0.82-1.57) | 0.90 (0.65-1.25) | 1.92 (0.14-26.42) | 1.21 (0.95-1.54) | 0.94 (0.79-1.11) |
| 80 | 1.17 (0.83-1.65) | 0.89 (0.61-1.30) | 2.26 (0.07-72.53) | 1.19 (0.93-1.52) | 0.99 (0.84-1.17) |
| p_nonlinearity_ | 0.58 | 0.86 | 0.79 | 0.10 | 0.27 |

**REFRENCES**

1. Perez-Cornago, A., et al., *Examination of potential novel biochemical factors in relation to prostate cancer incidence and mortality in UK Biobank.* Br J Cancer, 2020. **123**(12): p. 1808-1817.

2. Welsh, P., et al., *Lipoprotein(a) and cardiovascular disease: prediction, attributable risk fraction, and estimating benefits from novel interventions.* Eur J Prev Cardiol, 2020.

3. Zhang, M., et al., *Lipoprotein(a) and cardiovascular death in oldest-old (≥80 years) patients with acute myocardial infarction: A prospective cohort study.* Atherosclerosis, 2020. **312**: p. 54-59.

4. Cao, Y.X., et al., *Lipoprotein(a) and Cardiovascular Outcomes in Patients with Previous Myocardial Infarction: A Prospective Cohort Study.* Thromb Haemost, 2021.

5. Heinrich, N.S., et al., *Lipoprotein(a)and renal function decline, cardiovascular disease and mortality in type 2 diabetes and microalbuminuria.* J Diabetes Complications, 2020.

6. Golledge, J., et al., *Association of Serum Lipoprotein (a) With the Requirement for a Peripheral Artery Operation and the Incidence of Major Adverse Cardiovascular Events in People With Peripheral Artery Disease.* J Am Heart Assoc, 2020. **9**(6): p. e015355.

7. Arsenault, B.J., et al., *Association of Long-term Exposure to Elevated Lipoprotein(a) Levels with Parental Life Span, Chronic Disease-Free Survival, and Mortality Risk: A Mendelian Randomization Analysis.* JAMA Netw Open, 2020.

8. Liu, H.H., et al., *Association of lipoprotein(a) levels with recurrent events in patients with coronary artery disease.* Heart, 2020. **106**(16): p. 1228-1235.

9. Roth, C., et al., *Lipoprotein(a) plasma levels are not associated with survival after acute coronary syndromes: An observational cohort study.* PLoS ONE, 2020. **15**(1).

10. Xu, N., et al., *Impact of Lipoprotein(a) on Long-Term (Mean 6.2 Years) Outcomes in Patients With Three-Vessel Coronary Artery Disease.* Am J Cardiol, 2020. **125**(4): p. 528-533.

11. Zhang, Y., et al., *Lipoprotein (a) predicts recurrent worse outcomes in type 2 diabetes mellitus patients with prior cardiovascular events: A prospective, observational cohort study.* Cardiovasc Diabetol, 2020. **19**(1).

12. Gencer, B., et al., *Prognostic value of elevated lipoprotein(a) in patients with acute coronary syndromes.* Eur J Clin Invest, 2019. **49**(7).

13. O’Donoghue, M.L., et al., *Lipoprotein(a), PCSK9 inhibition, and cardiovascular risk insights from the FOURIER trial.* Circulation, 2019. **139**(12): p. 1483-1492.

14. Langsted, A., P.R. Kamstrup, and B.G. Nordestgaard, *High lipoprotein(a) and high risk of mortality.* Eur Heart J, 2019. **40**(33): p. 2760-2770.

15. Akinyemiju, T., et al., *Pre-diagnostic biomarkers of metabolic dysregulation and cancer mortality.* Oncotarget, 2018. **9**(22): p. 16099-16109.

16. Feng, Z., et al., *Association of lipoprotein(a) with long-term mortality following coronary angiography or percutaneous coronary intervention.* Clin Cardiol, 2017. **40**(9): p. 674-678.

17. Bajaj, A., et al., *Lipoprotein(a) and risk of myocardial infarction and death in chronic kidney disease findings from the CRIC study (Chronic renal insufficiency cohort).* Arterioscler Thromb Vasc Biol, 2017. **37**(10): p. 1971-1978.

18. Zewinger, S., et al., *Relations between lipoprotein(a) concentrations, LPA genetic variants, and the risk of mortality in patients with established coronary heart disease: a molecular and genetic association study.* Lancet Diabetes Endocrinol, 2017. **5**(7): p. 534-543.

19. Waldeyer, C., et al., *Lipoprotein(a) and the risk of cardiovascular disease in the European population: Results from the BiomarCaRE consortium.* Eur Heart J, 2017. **38**(32): p. 2490-2498.

20. Katzke, V.A., et al., *Blood lipids and lipoproteins in relation to incidence and mortality risks for CVD and cancer in the prospective EPIC-Heidelberg cohort.* BMC Med, 2017. **15**(1).

21. Onat, A., et al., *Lipoprotein(a) level and MIF gene variant predict incident metabolic syndrome and mortality.* J Invest Med, 2016. **64**(2): p. 392-399.

22. Séguro, F., et al., *Real life validation of the European Atherosclerosis Society Consensus Panel lipoprotein(a) threshold of 50 mg/dL.* Int J Cardiol, 2016. **221**: p. 537-538.

23. Zhao, Y., et al., *Cardiovascular Disease, Mortality Risk, and Healthcare Costs by Lipoprotein(a) Levels According to Low-density Lipoprotein Cholesterol Levels in Older High-risk Adults.* Clin Cardiol, 2016. **39**(7): p. 413-420.

24. Nomikos, T., et al., *Hierarchical modelling of blood lipids' profile and 10-year (2002-2012) all cause mortality and incidence of cardiovascular disease: The ATTICA study.* Lipids Health Dis, 2015. **14**(1).

25. Patterson, C.C., et al., *Which biomarkers are predictive specifically for cardiovascular or for non-cardiovascular mortality in men? Evidence from the Caerphilly Prospective Study (CaPS).* Int J Cardiol, 2015. **201**: p. 113-118.

26. Sawabe, M., et al., *Low lipoprotein(a) concentration is associated with cancer and all-cause deaths: A population-based cohort study (the jms cohort study).* PLoS ONE, 2012. **7**(4).

27. Qi, Q., et al., *Genetic variants, plasma lipoprotein(a) levels, and risk of cardiovascular morbidity and mortality among two prospective cohorts of type 2 diabetes.* Eur Heart J, 2012. **33**(3): p. 325-34.

28. Collaboration, E.R.F., *Lipoprotein (a) concentration and the risk of coronary heart disease, stroke, and nonvascular mortality.* 2009.

29. Solfrizzi, V., et al., *All-cause mortality and competing risks of fatal and nonfatal vascular events in the Italian longitudinal study on aging: impact of lipoprotein(a).* Rejuvenation Res, 2009. **12**(6): p. 395-402.

30. Chien, K.L., et al., *Lipoprotein(a) and cardiovascular disease in ethnic Chinese: The Chin-Shan community cardiovascular cohort study.* Clin Chem, 2008. **54**(2): p. 285-291.

31. D'Angelo, A., et al., *Lipoprotein(a), fibrinogen and vascular mortality in an elderly northern Italian population.* Haematologica, 2006. **91**(12): p. 1613-1620.

32. Hernández, C., et al., *Lipoprotein(a) as a risk factor for cardiovascular mortality in type 2 diabetic patients: A 10-year follow-up study.* Diabetes Care, 2005. **28**(4): p. 931-933.

33. Ariyo, A.A., C. Thach, and R. Tracy, *Lp(a) Lipoprotein, Vascular Disease, and Mortality in the Elderly.* New Engl J Med, 2003. **349**(22): p. 2108-2115.

34. Ahlbeckglader, C., et al., *Is lipoprotein(a) a predictor for survival in patients with established coronary artery disease? Results from a prospective patient cohort study in northern Sweden.* J Intern Med (GBR), 2002. **252**(1): p. 27-35.

35. Iliescu, E.A., et al., *Apolipoprotein(a) phenotype and lipoprotein(a) level predict peritoneal dialysis patient mortality.* Peritoneal Dial Int, 2002. **22**(4): p. 492-499.

36. Zairis, M.N., et al., *The impact of plasma levels of C-reactive protein, lipoprotein (a) and homocysteine on the long-term prognosis after successful coronary stenting: The global evaluation of new events and restenosis after stent implantation study.* J Am Coll Cardiol, 2002. **40**(8): p. 1375-1382.

37. Lundstam, U., et al., *Serum lipids, lipoprotein(a) level, and apolipoprotein(a) isoforms as prognostic markers in patients with coronary heart disease.* J Intern Med (GBR), 2002. **251**(2): p. 111-118.

38. Cheng, S.W.K. and A.C.W. Ting, *Lipoprotein (a) level and mortality in patients with critical lower limb ischaemia.* Eur J Vasc Endovasc Surg, 2001. **22**(2): p. 124-129.

39. Gerdes, L.U., et al., *The apolipoprotein ε4 allele determines prognosis and the effect on prognosis of simvastatin in survivors of myocardial infarction: A substudy of the Scandinavian Simvastatin Survival Study.* Circulation, 2000. **101**(12): p. 1366-1371.

40. Shlipak, M.G., et al., *Estrogen and progestin, lipoprotein (a), and the risk of recurrent coronary heart disease events after menopause.* Jama, 2000. **283**(14): p. 1845-1852.

41. Koda, Y., et al., *Lipoprotein(a) is a predictor for cardiovascular mortality of hemodialysis patients.* Kidney Int Suppl, 1999. **56**(71): p. S251-S253.

42. Ohashi, H., et al., *Lipoprotein(a) as a risk factor for coronary artery disease in hemodialysis patients.* Kidney Int Suppl, 1999. **56**(71): p. S242-S244.

43. Stubbs, P.J., et al., *Lipoprotein(a) as a risk predictor for cardiac mortality in patients with acute coronary syndromes.* Eur Heart J, 1998. **19**(9): p. 1355-1364.

44. Bigazzi, F., et al., *Gender difference in lipoprotein(a) concentration as a predictor of coronary revascularization in patients with known coronary artery disease.* Biochim Biophys Acta Mol Cell Biol Lipids, 2021. **1866**(3).

45. Hishikari, K., et al., *Usefulness of Lipoprotein (a) for Predicting Outcomes After Percutaneous Coronary Intervention for Stable Angina Pectoris in Patients on Hemodialysis.* Am J Cardiol, 2020. **136**: p. 32-37.

46. Matsushita, K., et al., *Impact of serum lipoprotein (a) level on coronary plaque progression and cardiovascular events in statin-treated patients with acute coronary syndrome: a yokohama-acs substudy.* J Cardiol, 2020. **76**(1): p. 66-72.

47. Dewa Gde Dwi Sumarjaya, I., I. Ketut Badjra Nadha, and A.A.W. Lestari, *High lipoprotein(a) levels as a predictor of major adverse cardiovascular events inhospitalized-acute myocardial infarction patients.* Vasc Health Risk Manage, 2020. **16**: p. 125-132.

48. Xu, N., et al., *Lipoprotein(a) levels are associated with coronary severity but not with outcomes in Chinese patients underwent percutaneous coronary intervention.* Nutr Metab Cardiovasc Dis, 2020. **30**(2): p. 265-273.

49. Shitara, J., et al., *Impact of lipoprotein (a) levels on long-term outcomes in patients with coronary artery disease and left ventricular systolic dysfunction.* Circ J, 2019. **83**(5): p. 1047-1053.

50. Sanchez Munoz-Torrero, J.F., et al., *Lipoprotein (a) levels and outcomes in stable outpatients with symptomatic artery disease.* Atherosclerosis, 2018. **276**: p. 10-14.

51. Fogacci, F., et al., *Serum lipoprotein(a) level as long-term predictor of cardiovascular mortality in a large sample of subjects in primary cardiovascular prevention: data from the Brisighella Heart Study.* Eur J Intern Med, 2017. **37**: p. 49-55.

52. Xie, H., et al., *Long-Term Prognostic Value of Lipoprotein(a) in Symptomatic Patients With Nonobstructive Coronary Artery Disease.* Am J Cardiol, 2017. **119**(7): p. 945-950.

53. Konishi, H., et al., *Plasma lipoprotein(a) predicts major cardiovascular events in patients with chronic kidney disease who undergo percutaneous coronary intervention.* Int J Cardiol, 2016. **205**: p. 50-53.

54. Mitsuda, T., et al., *Lipoprotein (a) levels predict adverse vascular events after acute myocardial infarction.* Heart and vessels, 2016. **31**(12): p. 1923-1929.

55. Konishi, H., et al., *Impact of Lipoprotein(a) as residual risk on long-term outcomes in patients after percutaneous coronary intervention.* Am J Cardiol, 2015. **115**(2): p. 157-160.

56. Cai, A., et al., *Baseline LDL-C and Lp(a) elevations portend a high risk of coronary revascularization in patients after stent placement.* Dis Markers, 2013. **35**(6): p. 857-862.

57. Zhou, J., et al., *Association between Lipoprotein (a) level on admission and the incidence of subsequent cardiovascular events in patients with acute coronary syndrome.* Int J Cardiol, 2012. **158**(3): p. 464-6.

58. Ikenaga, H., et al., *Usefulness of lipoprotein (a) for predicting progression of non-culprit coronary lesions after acute myocardial infarction.* Circ J, 2011. **75**(12): p. 2847-2852.

59. Boras, J., et al., *Lipoprotein(a) predicts progression of carotid artery intima-media thickening in patients with type 2 diabetes: A four-year follow-up.* Wien Klin Wochenschr, 2010. **122**(5-6): p. 159-164.

60. Nicholls, S.J., et al., *Lipoprotein(a) levels and long-term cardiovascular risk in the contemporary era of statin therapy.* J Lipid Res, 2010. **51**(10): p. 3055-3061.

61. Igarashi, Y., et al., *Predictors of adverse long-term outcome in acute myocardial infarction patients undergoing primary percutaneous transluminal coronary angioplasty - With special reference to the admission concentration of lipoprotein (a).* Circ J, 2003. **67**(7): p. 605-611.

62. Longenecker, J.C., et al., *Small apolipoprotein(a) size predicts mortality in end-stage renal disease: The CHOICE study.* Circulation, 2002. **106**(22): p. 2812-2818.

63. Cristóbal, C., et al., *Levels of lipoprotein(a) in cardiac transplant recipients.* Transplant Proc, 1999. **31**(6): p. 2552-2553.

64. Skinner, J.S., et al., *Serum Lp (a) lipoprotein concentration is not associated with clinical and angiographic outcome five years after coronary artery bypass graft surgery.* Heart, 1997. **78**(2): p. 131-135.

65. Goldwasser, P., et al., *Prealbumin and lipoprotein(a) in hemodialysis: Relationships with patient and vascular access survival.* AM J KIDNEY DIS, 1993. **22**(1): p. 215-225.

66. Schwaiger, J.P., et al., *Carotid plaques and their predictive value for cardiovascular disease and all-cause mortality in hemodialysis patients considering renal transplantation: A decade follow-up.* Am. J. Kidney Dis., 2006. **47**(5): p. 888-897.

67. Hocher, B., et al., *Different impact of biomarkers as mortality predictors among diabetic and nondiabetic patients undergoing hemodialysis.* J Am Soc Nephrol, 2003. **14**(9): p. 2329-2337.

68. Zimmermann, J., et al., *Inflammation enhances cardiovascular risk and mortality in hemodialysis patients.* Kidney international, 1999. **55**(2): p. 648-658.

69. Koch, M., et al., *Apolipoprotein A, fibrinogen, age, and history of stroke are predictors of death in dialysed diabetic patients: A prospective study in 412 subjects.* NEPHROL DIAL TRANSPLANT, 1997. **12**(12): p. 2603-2611.

70. Cressman, M.D., et al., *Lp (a) and premature mortality during chronic hemodialysis treatment.* Chemistry and physics of lipids, 1994. **67**: p. 419-427.

71. Cressman, M.D., et al., *Lipoprotein(a) Is an Independent Risk Factor for Cardiovascular-Disease in Hemodialysis-Patients.* Circulation, 1992. **86**(2): p. 475-482.

72. Haffner, S.M., et al., *Lack of association between lipoprotein (a) concentrations and coronary heart disease mortality in diabetes: The Wisconsin Epidemiologic Study of Diabetic Retinopathy.* METAB CLIN EXP, 1992. **41**(2): p. 194-197.
